# The genetic footprint of the European Roma diaspora: evidence from the Balkans to the Iberian Peninsula

**DOI:** 10.1007/s00439-025-02735-z

**Published:** 2025-03-17

**Authors:** Giacomo Francesco Ena, Aaron Giménez, Annabel Carballo-Mesa, Petra Lišková, Marcos Araújo Castro e Silva, David Comas

**Affiliations:** 1 Institut de Biologia Evolutiva (CSIC-UPF), Universitat Pompeu Fabra, Departament de Medicina i Ciències de la Vida, Barcelona, Spain; 2 Facultat de Sociologia, Universitat Autònoma de Barcelona, Barcelona, Spain; 3 Facultat de Geografia i Història, Universitat de Barcelona, Barcelona, Spain; 4 Department of Paediatrics and Inherited Metabolic Disorders, First Faculty of Medicine, Charles University and General University Hospital in Prague, Prague, Czech Republic; 5 Department of Ophthalmology, First Faculty of Medicine, Charles University and General University Hospital in Prague, Prague, Czech Republic

## Abstract

The Roma people have a complex demographic history shaped by their recent dispersal from a South Asian origin into Europe, accompanied by continuous population bottlenecks and gene flow. After settling in the Balkans around 1,000 years ago, the Roma gradually dispersed across Europe, and approximately 500 years ago, they established in the Iberian Peninsula what is now one of the largest Roma populations in Western Europe. Focusing specifically on the Iberian Roma, we conducted the most comprehensive genome-wide analysis of European Roma populations to date. Using allele frequency and haplotype-based methods, we analysed 181 individuals to investigate their genetic diversity, social dynamics, and migration histories at both continental and local scales. Our findings demonstrate significant gene flow from populations encountered during the Roma’s dispersal and confirm their South Asian origins. We show that, between the 14th and 19th centuries, the Roma spread westward from the Balkans in various waves, with multiple admixture events. Furthermore, our findings refute previous hypotheses of a North African dispersal route into Iberia and genetic connections to Jewish populations. The Iberian Roma exhibit ten times greater genetic differentiation compared to non-Roma Iberians, indicating significant regional substructure. Additionally, we provide the first genetic evidence of assortative mating within Roma groups, highlighting distinct mating patterns and suggesting a gradual shift towards increased integration with non-Roma individuals. This study significantly enhances our understanding of how demographic history and complex genetic structure have shaped the genetic diversity of Roma populations, while also highlighting the influence of their evolving social dynamics.

**Supplementary Information:**

The online version contains supplementary material available at 10.1007/s00439-025-02735-z.

## Introduction

The Romani population, often inaccurately referred to as “Gypsies”, is recognised as Europe’s largest transnational ethnic minority (O’Nions 2016). Conservative estimates place the Romani population at approximately 10 million, though, without a formal census, this number is likely an underestimation (Bernát and Messing 2016). While Roma groups share a common identity and cultural traditions, their linguistic diversity is notable (Matras 2002). Our understanding of the Roma’s historical origins has been derived from a combination of linguistic studies, historical records, and more recent genetic research. Previous investigations trace their roots to South Asia, particularly the Punjab and Kashmir regions, as supported by both historical sources (Iovita and Schurr 2004; Kenrick 2007) and genetic findings (Kalaydjieva et al. 2001; Mendizabal et al. 2012; Moorjani et al. 2013; Martínez-Cruz et al. 2016; Font-Porterias et al. 2019; Ena et al. 2022).

Embarking on their diaspora from South Asia, the Roma travelled through what is now Afghanistan and various regions of Western Asia and the Caucasus, such as Iran, Armenia, and Anatolia, before entering Europe through the Balkans during the Middle Ages (Fraser 1992; Hancock 2006; Kenrick 2007). Upon their arrival, they faced a mixed reception that escalated into persecution and slavery, conditions that persisted in several European kingdoms until the 19th century (Brearley 2001). This tragic history culminated in the Nazi genocide during the Second World War (Lewy 2000; Kenrick and Puxon 2009). Although they are now recognised as full-fledged citizens within the European Union, the Roma community continues to grapple with discrimination, high unemployment rates, pervasive poverty, and significant health disparities (O’Nions 2011; Parekh and Rose 2011; Ivanov and Kagin 2014; Kajanova and Kmecova 2018).

Today, the Roma community is found throughout Europe, with the Iberian Peninsula hosting one of the largest Roma groups. Known by the endonym *Calé*, this group represents the westernmost edge of the Roma Diaspora within the continent. Historical accounts suggest that the Roma arrived on the Iberian Peninsula in the 15th century, with the earliest documentation in Zaragoza in 1425, having travelled from south-eastern Europe via a northern route (Pym 2007; Kenrick 2007; Sánchez 2022). There is speculation, based on oral traditions within some Roma communities, as well as historical interpretations of religious texts and the earlier belief that the Roma originated from Egypt, that some travellers may have reached the Iberian Peninsula through an alternative route via the Arabian Peninsula, North Africa, and the Strait of Gibraltar (Aparicio Gervás 2006; Hancock 2006; Pohoryles 2018), although no solid evidence supports this hypothesis.

The arrival of the Roma into Iberia coincided with a period of significant population and political turmoil, during which the Islamic rule of seven centuries was overthrown by Christian kingdoms. In the late 15th and early 16th centuries, these kingdoms persecuted, expelled, and forced religious conversions of Muslims and Jewish (Soyer 2007; Tartakoff 2012; Kimmel 2015; Carr 2017). This era was marked by considerable upheaval as Christian Spaniards, Jews, and Muslims coexisted for nearly a century. During this turbulent period, there may have been instances of genetic admixture between the Roma and these groups, who were eventually expelled in the 16th century, while the Roma were compelled to settle (Pym 2007; Abreu 2007; Sánchez 2022). Although initially tolerated, by the 16th century the first laws against the Roma were enacted in Spain and Portugal (Leblon 1985; Ortega 1994; Martínez Dhier 2007; Abreu 2007), restricting their freedom. This culminated in Spain with the “Gran Redada” (Great Round-up) on July 30, 1749, during which thousands of Roma individuals were arrested and imprisoned (Sánchez 2022). The 19th and 20th centuries saw significant internal movements within Spain and the broader peninsula, contributing to the displacement of many people, likely including the Roma (Bover and Velilla 1999; Silvestre 2005). Today, the Iberian Roma constitute the largest Roma population in Western Europe, estimated at nearly a million individuals, predominantly concentrated in the Andalusian region (Laparra 2007; Laparra et al. 2007). Despite this, they continue to suffer from socioeconomic inequities compared to non-Roma individuals (Tarnovschi et al. 2012; La Parra-Casado et al. 2018; Mendes and Magano 2022).

Previous genetic studies on the Roma population of the Iberian Peninsula have primarily focused on uniparental markers (Gusmão et al. 2008; Mendizabal et al. 2011; Gómez-Carballa et al. 2013; García-Fernández et al. 2020; Aizpurua-Iraola et al. 2022) with a smaller portion dedicated to the X chromosome, genome-wide array-data (Pereira et al. 2012; Font-Porterias et al. 2019), and complete genomes (Bianco et al. 2020). These studies have generally centred on broad historical inquiries, origins, and differentiation of European Roma on a large scale rather than specifically targeting Iberian Roma groups. Despite limitations due to partial genome data and small sample sizes, findings have shown that Iberian Roma share a common origin with other European Roma groups (Mendizabal et al. 2012; Moorjani et al. 2013; Martínez-Cruz et al. 2016; Font-Porterias et al. 2019), possess unique uniparental haplotypes (Gusmão et al. 2008; Gómez-Carballa et al. 2013; Aizpurua-Iraola et al. 2022), and appear to be part of the first out-of-Balkan migration along with Central and Northern European Roma (Mendizabal et al. 2011). This westernmost expansion of the Roma in Europe exhibits the highest proportion of European-like ancestral components compared to other Roma populations and appears to display geographical substructure within the Iberian Peninsula (Font-Porterias et al. 2019). However, these analyses have not fully explored the internal diversity of Roma groups, their heterogeneity (both within Iberia and across Europe), or how socio-cultural customs have shaped gene flow and population admixture within the Roma community. Additionally, the role of assortative mating in shaping the distribution of genetic variation among these populations has yet to be investigated.

To address these previous limitations, and as a part of a Roma community-driven initiative in collaboration with the FAGiC (Federation of Roma Associations of Catalonia), we have analysed genome-wide data from 105 Iberian Roma volunteers, comparing them with other Roma and non-Roma groups. Our genomic approach focuses on: (i) the internal genetic structure of Roma and its correlation with geography; (ii) the relationships between Iberian Roma and other non-Roma populations to explore possible contacts during the period of population upheaval following the Roma’s arrival on the Iberian Peninsula; (iii) estimation of the admixture events in the Iberian Roma before and after their arrival to the Iberian Peninsula; and (iv) the assessment of endogamy patterns (understood as marriage within the population or community) and assortative mating of the European Roma. Our study provides a detailed analysis of the intricate population history of the Iberian Roma, offering insights from both micro-geographical and large-scale perspectives on the population history of European Roma.

## Results

### Genetic distinctiveness of the Roma within the European context

**Fig. 1:**
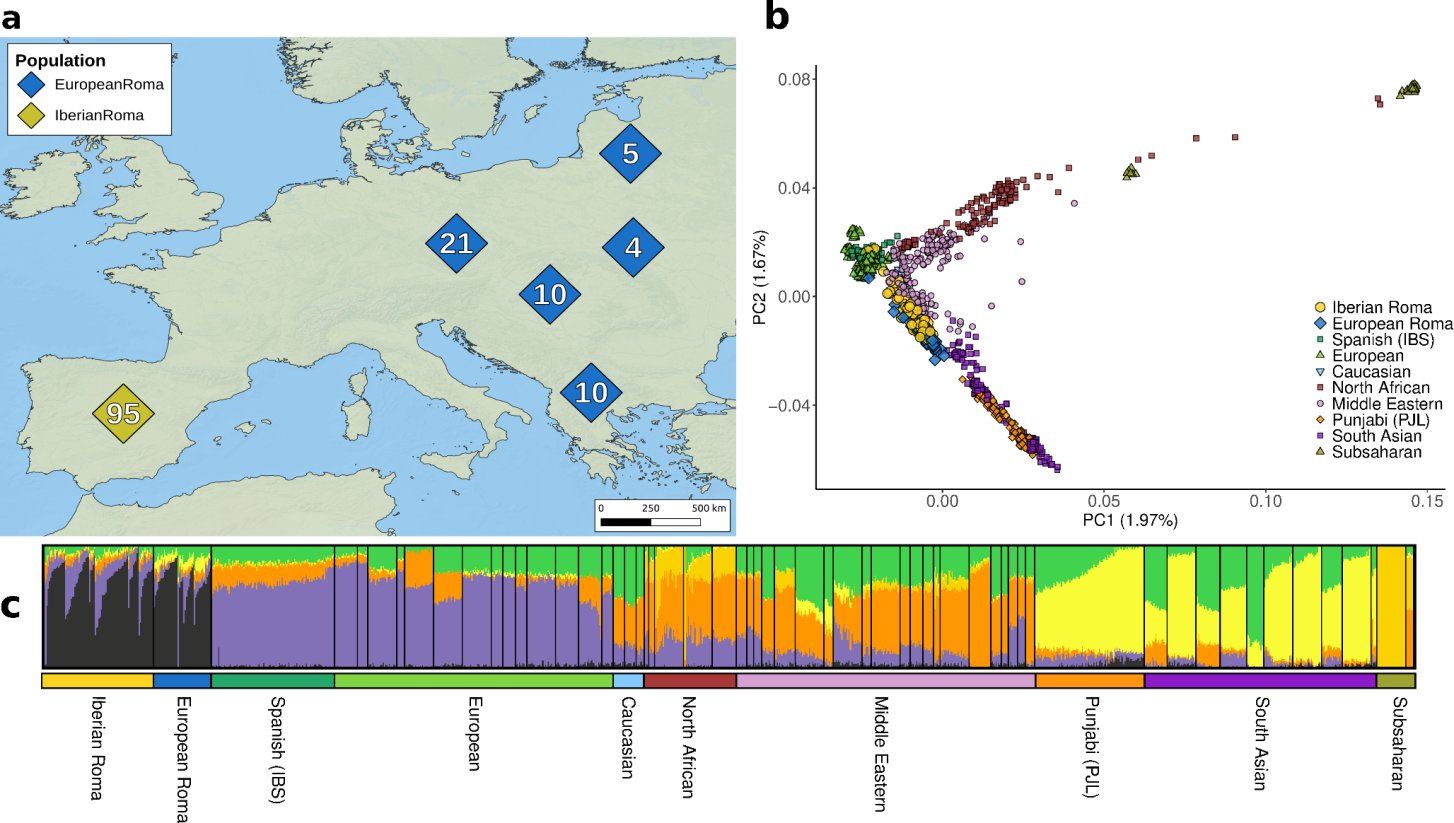
Genetic population structure of the European Roma groups. (**a**) Distribution and sample sizes of Roma populations included in the present study. (**b**) Principal component analysis (PCA) of all samples analysed in this study. IBS and PJL stand for Iberian Spanish and Punjabi from the 1000 Genomes Project, respectively. (**c**) ADMIXTURE results showing the lowest cross-validation error (K = 6)

The genetic relationships between Roma individuals and other populations were assessed through Principal Component Analysis (PCA) and ADMIXTURE analyses. The plot of the first two PC components (Fig. 1b) shows that Iberian Roma, along with other European Roma groups, cluster between European and Indian populations, with minimal overlap with other groups (the first four principal components are shown in Fig. 1b and Supplementary Fig. 1). This pattern is consistent with previous genetic studies (Mendizabal et al. 2012; Ceballos et al. 2018; Font-Porterias et al. 2019; Bianco et al. 2020). The ADMIXTURE analysis at K = 4 reveals populations clustering by continental origin, with the Roma forming a distinct group, showing with two main components linked to European and South Asian populations (Supplementary Fig. 2). At the lowest cross-validation error, K = 6 (Supplementary Fig. 3), a Roma-specific component is highlighted, ranging from approximately 30–80% (Fig. 1c and S2).

To investigate gene flow patterns from populations encountered during the Roma diaspora, we analysed allele sharing using the outgroup *f3*-statistics and *f4-*statistics. In the *f3* outgroup test, the non-Roma European populations showed the highest *f3* values (Supplementary Fig. 4a-c), followed by West Asian and South Asian groups, with African populations showing the least affinity, irrespective of the Roma subgroup (Supplementary Fig. 4a-c). The *f4* tests showed that Roma are genetically closer to European groups than to South Asian ones (Supplementary Fig. 5). Among European groups, Iberian Roma were more closely related to the Basques (Supplementary Fig. 6a), while Czech Roma were genetically closer to Central Europeans (CEU) (Supplementary Fig. 6a). When examining proximity to South Asian groups, both Iberian and Czech Roma showed divergence from southern and western Indian populations (STU, BEB, ITU) and closer genetic affinity with Pakistani and northwestern Indian groups (Supplementary Fig. 6b), supporting the hypothesis of a Punjabi origin of the Roma diaspora (Mendizabal et al. 2011; Pamjav et al. 2011; Rai et al. 2012; Martínez-Cruz et al. 2016).

We further explored the genetic relatedness among populations using FineStructure analysis, which examines haplotype similarities. This analysis revealed that most Roma individuals cluster within a single macrobranch, with the exception of two Iberian individuals clustering with non-Roma Iberians (IBS) and one Hungarian Vlax individual clustering with the general Hungarian population. These Roma individuals were not included in further dating analyses to avoid estimation biases (Supplementary Fig. 7a-c). Within the Roma macrobranch, a geographical structure emerged, with distinct clusters for Iberian, Czech, and Macedonian Roma, while other European Roma clustered together, likely due to their small sample sizes (Supplementary Fig. 7a-c). For subsequent analyses, we grouped all Iberian Roma samples into one Recipient cluster (*IberianRoma*) and all other European Roma samples into another Recipient cluster (*EuropeanRoma*).

**Fig. 2:**
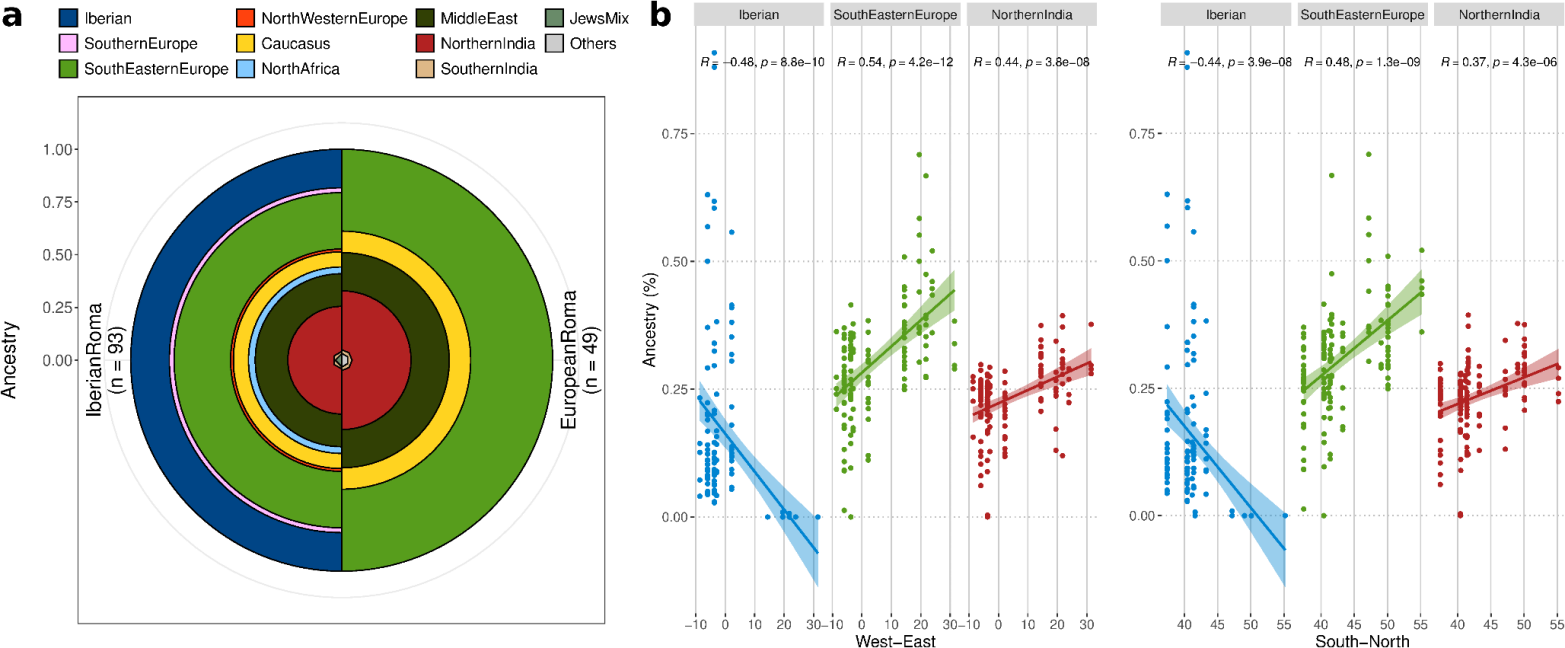
Ancestry proportions in Roma and geographic correlations. (**a**) Inferred proportions of shared ancestry among Iberian (*N* = 93) and European Roma (*N* = 49) clusters using the NNLS method. Roma individuals that clustered outside the Roma branch in the FineStructure dendrogram were not included in this analysis. (**b**) Pearson’s correlation tests for the three main ancestral components (Iberian, Southeastern European, and Indian) derived from the NNLS analysis, plotted against longitude and latitude for the Roma

Ancestry profiles derived from NNLS analysis showed that Roma individuals primarily shared haplotypes with European clusters, particularly *Balkan* and *CentralEurope*, followed by West and South Asian clusters, though the order varied (Supplementary Fig. 8). Minor components included other populations with negligible contributions. To simplify the interpretation of the NNLS results, we grouped the Donor clusters (Supplementary Table 1a) into 14 geographical macro-regions. Both Roma clusters show similar Donor compositions, although Iberian Roma showed a higher non-Roma Iberian ancestry and traces of southern European and North African ancestry, which were absent in other European Roma groups (Fig. 2a). We further divided the Iberian Roma in five subclusters and the European Roma in three subclusters (Supplementary Table 1b) for finer-scale analysis. Among the Iberian Roma subclusters, the European component ranged from 42 to 70%, while the South Asian component varied from 13 to 27%. Notably, the *IberianRomaSouthEast* subcluster exhibited the highest European and lowest South Asian percentages, with the other subclusters displaying more similar percentages (Supplementary Fig. 9a-b).

To further examine ancestry proportions, we estimated population-level averages from the NNLS results rather than using cluster averages. These findings revealed moderate differences, suggesting lower heterogeneity among Iberian populations compared to the clusters, and also among European populations. The primary distinction between Iberian Roma and other European Roma lies in the presence of Iberian and North African components, along with smaller South Asian and Southeastern European components (Supplementary Fig. 10a-b).

In summary, the Roma populations analysed exhibit genetic profiles with varying proportions of West Eurasia and South Asia ancestry, shaped by geographic factors. Pearson’s correlation tests on the NNLS ancestry components by macro-region revealed significant correlations between longitude and several ancestral components (Supplementary Table 2a-b; Supplementary Fig. 11). Specifically, there is an east-to-west and south-to-north decrease in Southeastern European and Northern Indian components among European Roma, reflecting greater gene flow with non-Roma groups outside the Balkans. Conversely, an east-to-west increase in Iberian and North African components was observed among Iberian Roma, indicating higher gene flow with non-Roma Iberians (Fig. 2b).

### Multiple events of admixture in European Roma groups

**Fig. 3:**
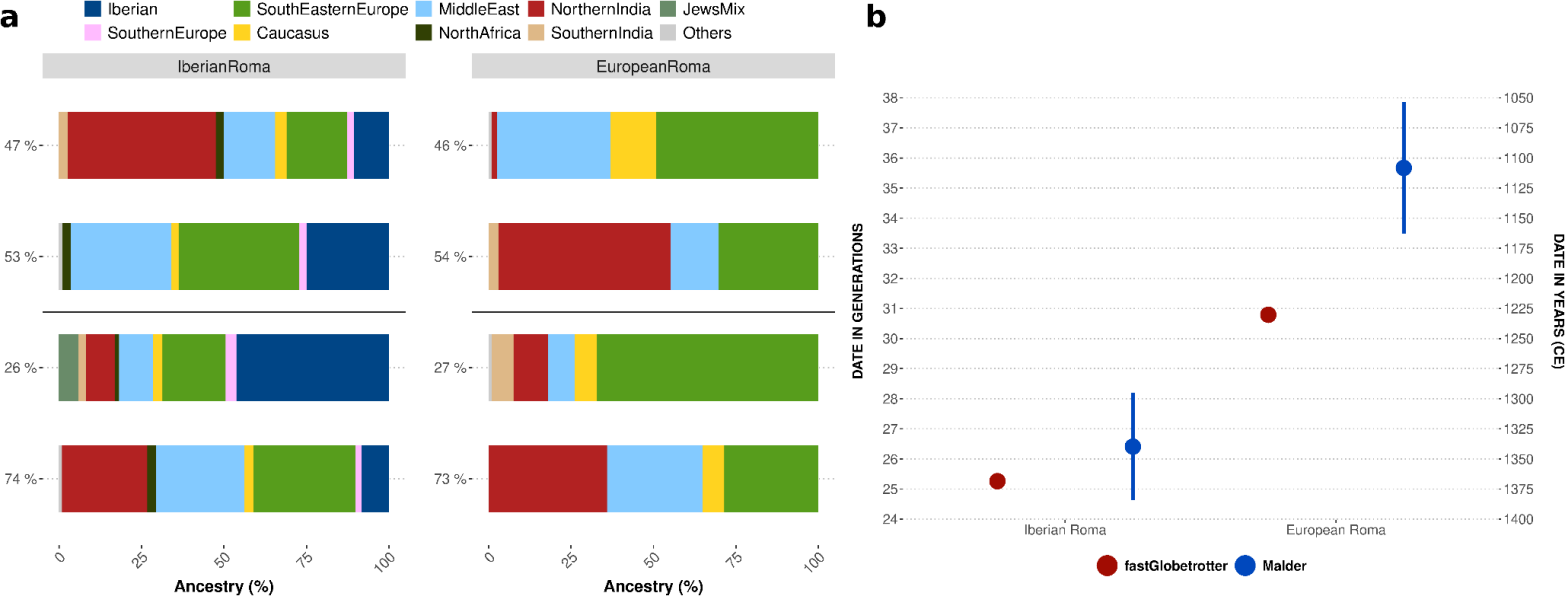
Admixture events in Roma history. (**a**) Admixture events inferred by fastGLOBETROTTER, showing relative ancestry proportions grouped into macro-regions for each source of the two inferred admixture events. The plot displays recent admixture events at the bottom and older events at the top, separately for Iberian Roma (*n* = 93) and European Roma (*n* = 49). (**b**) Comparison of inferred admixture events between the fastGLOBETROTTER (representing older events) and MALDER dating methods. CE = Common Era

To estimate the timing of the major admixture events in the Roma populations, we used two methods based on an admixture pulse model. To explore admixture dynamics across various geographical scales, we conducted fastGLOBETROTTER analyses on the *IberianRoma* and *EuropeanRoma* clusters (Supplementary Tables 3 and Supplementary Fig. 12a-b), followed by analyses of the Iberian and European Roma subclusters (Supplementary Table 3a and Fig. 12c-d). The fastGLOBETROTTER analysis identified multiple waves of admixture (Fig. 3a), while MALDER detected only a single event for each significant test. For the *EuropeanRoma* cluster, two significant admixture events were identified (Fig. 3, Supplementary Table 3a-b). The first occurred approximately 30 ± 0.49 generations ago (GA) (1218 to 1243 CE, assuming 25 years per generation), involving two nearly equal ancestry sources: a major source with over 50% South Asian ancestry and a minor source predominantly consisting of Southeastern European, Caucasus, and Middle Eastern/West Eurasian ancestries. The second event, dated about 10.06 ± 1.06 GA (1722 to 1775 CE), had a major source contributing 73%, characterised by a blend of the earlier sources, evenly distributed among Southeastern European, Caucasus-Middle Eastern, and South-Asian groups. The minor source in this recent event was primarily European, with smaller contributions from West and South Asian ancestries. For the Iberian Roma cluster (Fig. 3, Supplementary Table 3a), two admixture events were also identified. The first occurred approximately 25 ± 0.21 GA (1363 to 1374 CE), with two sources: one comprising 64% European (mainly Iberian) and Caucasus-Middle East components, and the other an equal mix of South Asian and European/West Asian ancestries, including a small 2% North African component. The second event, dated to about 5 ± 0.29 GA (1864 to 1878 CE), had a major source showing a balanced contribution from European, Caucasus-Middle Eastern, and South Asian ancestries, while the minor source was predominantly West Eurasian, with a small South Asian component.

The key differences between the Iberian Roma and European Roma clusters were the presence of Iberian, North African, and Southern European components in the Iberian Roma, compared to a much larger Central and Southeastern European components in the European Roma. Additionally, the minor source in the recent admixture event of Iberian Roma included a JewsMix ancestry component, which comprises Jewish individuals from Poland, Turkey, and Morocco. This component, absent in European Roma, may indicate gene flow from North African or Jewish groups.

MALDER’s estimates, which identified single admixture events for each reference pair, also revealed several statistically significant results (Supplementary Table 4). For the *EuropeanRoma*, using *Punjabi1* and *Balkan* clusters as references, the estimated date was 35.67 ± 2.18 (1001 to 1215 CE). For the *IberianRoma*, using *Punjabi1* and *IBS* as references, the estimated date was 26.41 ± 1.78 GA (1253 to 1427 CE), while using *Punjabi1* and *Balkan* clusters produced a date of 28.81 ± 2.22 GA (1171 to 1389 CE). These *IberianRoma* estimates overlap with those from fastGLOBETROTTER but have larger standard errors (Fig. 3b). To further trace the earliest Roma migrations and their dispersion routes from South Asia, we used MALDER to infer admixture dates between incoming Roma and local populations. Using *Punjabi1* as a proxy for proto-Roma ancestry, we tested whether the Iberian Roma could have been formed through admixture events with various populations along the proposed dispersion path. As expected from a westward dispersal originating in South Asia, the earliest date (36.49 ± 2.22 GA) was obtained for an admixture event between the *Punjabi1* and *Iranian* clusters (Supplementary Fig. 13). The dates inferred for other groups along the way were consistent with this hypothesis, showing more recent admixture dates along an east-to-west axis, with the latest date (25.23 ± 1.81 GA) corresponding to an admixture event between *Punjabi1* and *Basque* clusters (Supplementary Fig. 13). Both methods used to infer admixture dates identified sources consistent with the NNLS analysis, primarily composed of South Asian, Southeastern European, and Middle Eastern sources, with the addition of an Iberian source specifically in the Iberian Roma.

### Evolution of admixture dynamics over time

We investigated how admixture dynamics with non-Roma European populations have varied over time among different Roma groups by analysing the distribution patterns of European local ancestry segment sizes and the shared IBD segments within Roma groups and between Roma and non-Roma Europeans. We categorised these European segments based on their length, using time approximations from previous studies (Baharian et al. 2016; Harris et al. 2018; Castro e Silva et al. 2022) to infer when the haplotypes were formed (see Supplementary Note 1 and Supplementary Note 2). On average, the length of non-Roma European ancestral segments was similar across different Roma groups, ranging from 15.7 to 22.3 Mb (Supplementary Fig. 14a). When analysing the number of non-Roma European segments by length category (Supplementary Fig. 14b), Iberian Roma, along with the Romungro from Hungary and Lithuanian Roma, exhibited an increased number of segments in the longer categories (representing more recent admixture events). In the shorter categories, there were fewer differences, or none, compared to other Roma groups. The average number of segments in most categories for Iberian Roma differed significantly compared to Czech Roma and the Romungro from Ukraine (Supplementary Table 5b). This suggests that the timing of admixture with non-Roma Europeans has varied across Roma groups, with a more recent increase observed in Iberian Roma over the last 200 years. This period corresponds to the end of the Roma slavery and the contemporary era (Greenberg 2010; Marushiakova and Popov 2010).

To further support the idea of changing levels of admixture over time, we analysed the distribution of shared IBD segments within Roma populations and between Roma and non-Roma populations. Iberian Roma consistently showed significantly lower levels of within-population shared IBD compared to other Roma groups (Supplementary Fig. 15, Supplementary Table 6a-b). Conversely, shared IBD between Roma and non-Roma groups did not significantly differ in most cases when comparing Iberian Roma to other Roma groups (Supplementary Table 7). This suggests that while Iberian Roma have experienced lower levels of endogamy, they have maintained similar levels of gene flow with local non-Roma populations compared to other Roma groups.

### Roma in the Iberian Peninsula exhibit high internal genetic structure

**Fig. 4:**
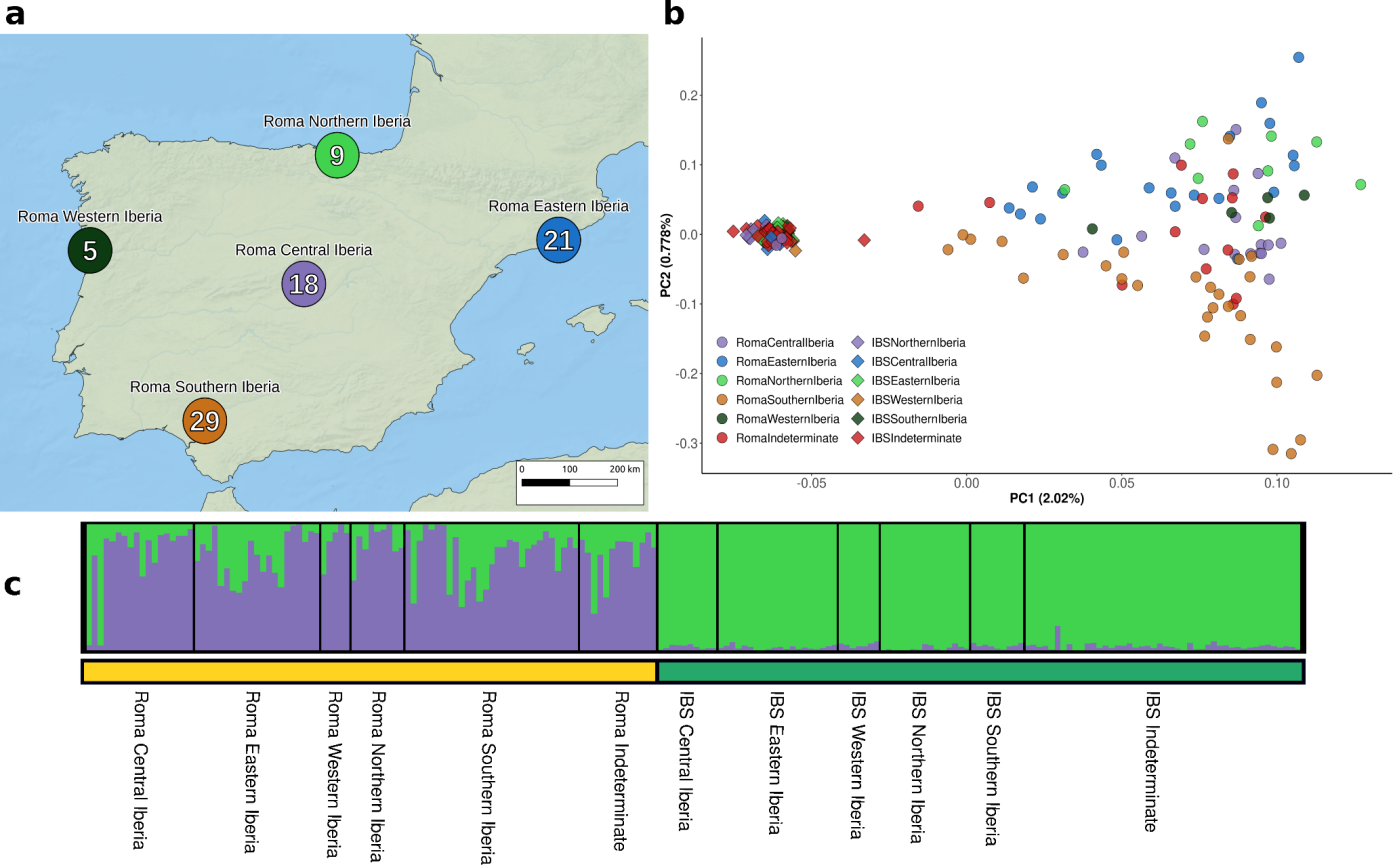
Population stratification of the Iberian Peninsula. (**a**) Distribution and size of Iberian Roma populations included in this study. Iberian Indeterminate population is not represented on the map due to insufficient geographic information. (**b**) Principal Component Analysis (PCA) using the Iberian dataset. (**c**) ADMIXTURE results showing the lowest cross-validation error (K = 2)

In addition to exploring the continental-scale genetic structure of European Roma, we aimed to investigate the genetic structure of Roma at a regional level. To this end, we assessed the genetic structure of Iberian samples (both Roma and non-Roma) using PCA and ADMIXTURE analyses, classifying the samples based on their geographic locations within the Iberian Peninsula (see Material and Methods) (Fig. 4a). The PCA plot reveals a clear distinction between Roma and non-Roma individuals (Fig. 4b). While non-Roma form a cohesive and distinct cluster, the Roma are more scattered across the plot, indicating regional substructure, as evidenced by the non-overlapping averages of PC1 and PC2 estimated for each Roma geographical group (Supplementary Fig. 16). The ADMIXTURE plot at the lowest cross-validation error K = 2, further differentiates Roma and non-Roma groups: Iberian non-Roma (IBS) show a single ancestral component, whereas Iberian Roma exhibit two components at varying frequencies across individuals (Fig. 4c). In contrast, the IBS display uniformity in their ancestral components even at K = 4, while the Roma show four components that are unevenly distributed (Supplementary Fig. 17; cross-validation plot in Supplementary Fig. 18).

To detect differences in ancestry profiles across Roma from different Iberian regions, we conducted an NNLS analysis, which showed that proportions are highly similar across all regions (Supplementary Fig. 19).

We formally tested the genetic structure of the Iberian samples through a series of AMOVA analyses (Supplementary Table 8). The analyses were conducted in four steps, including all individuals from the Iberian dataset except those classified as “Indeterminate”. When Iberian Roma and IBS were considered as a single group, significant genetic heterogeneity was detected between individuals (2.79%, *p* = 0.00001). When these two groups were analysed separately, 1.43% (*p* < 0.0001) of genetic heterogeneity was observed. To further explore internal heterogeneity within each group, we found that genetic heterogeneity within the Iberian Roma (1.07%, *p* = 0.0001) was more than ten times higher than that among Iberian non-Roma (0.08%, *p* = 0.0021). This finding was reinforced when Roma samples were grouped by region, showing significant heterogeneity (0.55%; *p* = 0.0001) between regions, while the regional heterogeneity in IBS is ten times lower (0.04%, *p* = 0.0019).

Finally, we estimated gene flow between Iberian Roma and IBS. A *f4* test was conducted to determine whether Iberian Roma individuals from different geographical regions share more alleles with non-Roma from the same regions. The results indicate that Roma groups share nearly equal numbers of alleles with all non-Roma groups, except for the IBS from Western Iberia (Supplementary Fig. 20a). Eastern Iberian Roma and *Indeterminate* Roma groups tend to show slightly greater genetic similarity with IBS, while comparisons involving IBS and Western and Central Iberian Roma often fall outside the significant threshold (Supplementary Fig. 20a).

To investigate interactions with Jewish and North African populations following the arrival of the Roma in the Iberian Peninsula in the 15th century, *f4* tests were conducted to analyse gene flow between these groups. The results indicate that Jews and North Africans share more alleles with the Iberian non-Roma than with the Iberian Roma, except for the Cochin Jews from India (Supplementary Fig. 20b-c). This may be due to the absence of direct gene flow between Roma and these populations, while the genetic proximity to Cochin Jews likely reflects the Indian origin of the Roma.

### Mating patterns in the Roma population: endogamy and ancestry-assortative mating

We analysed shared segments of IBD to understand demographic history and mating patterns within the Roma population. Compared to other global populations (Supplementary Fig. 21), Roma exhibit a high degree of internal IBD sharing, both in terms of segment size and number (Supplementary Tables 9 and 10). This level of sharing surpasses that observed in traditionally endogamous populations, such as the Cochin Jews, Brahui, Balochi, and Mozabite (Reich et al. 2009; Henn et al. 2012; Waldman et al. 2016), as well as Jews from Libya and Tunisia (Campbell et al. 2012). Iberian Roma share more IBD segments with Basque, Punjabi, Greek, and Iberian (IBS) populations; while European Roma share the most with Greek, Punjabi, Hungarian, and Czech populations. In general, Roma groups share more IBD segments with Greek than Punjabi, although Iberian Roma show lower levels of sharing with both (Supplementary Tables 9 and 10).

The inferred historical effective population size (*N*_*e*_) estimates for most Roma groups reveal a common pattern (Supplementary Fig. 22). *N*_*e*_ has remained low, gradually decreasing from around 50 to 8 generations ago (800–1800 CE), with the lowest *N*_*e*_ occurring between 7 and 29 generations ago (Supplementary Table 11). In contrast, reference populations such as Iberian, Czech, and Punjabi exhibit larger *N*_*e*_ values (Supplementary Fig. 22) and do not show a similar decline in *N*_*e*_ over time.

Genetic bottlenecks, like those experienced by the Roma, result in a higher number of shared ancestors over time and an increase in long runs of homozygosity (ROHs) (Ceballos et al. 2018). In populations where mating pairs share recent ancestors, such as the Roma, a higher number of long ROHs is expected, as they indicate recent endogamy. Conversely, many short ROHs can signal older bottlenecks followed by genetic admixture with different communities. Analysing the distribution of ROH lengths provides insight into the historical patterns of isolation and endogamy within the Roma population. To explore these patterns, we examined the length and number of ROH in our cohort (Supplementary Table 12) and found that Roma groups generally have more and longer ROHs compared to non-Roma reference populations (Supplementary Fig. 23). When analysing the average number of ROHs by length category, Roma groups exhibit fewer short ROHs but more medium-length ROHs compared to most populations (Supplementary Fig. 24a-b), with numbers comparable to South Asian groups such as Kalash and West Asian groups like the Bedouins in most categories (Supplementary Fig. 24a-d). These findings reinforce the notion that the Roma have higher levels of endogamy compared to non-Roma Europeans, primarily due to more recent isolation.

Lastly, to investigate the presence of ancestry-assortative mating within Roma populations, we applied the ANCESTOR method to the previously inferred local ancestry tracts, focusing on the proportions of European and South Asian ancestries. The results revealed that the inferred ancestries of mating pairs over the last generation showed significantly positive correlations in most Roma groups, deviating from the expectation under random mating (Supplementary Fig. 25a-b). This suggests that European and South Asian ancestry traits have influenced the mating patterns within the Roma population.

## Discussion

Our comprehensive genome-wide analysis of several European Roma groups reveals that the Roma population has a genetic profile composed of approximately two-thirds West Eurasian ancestry and one-third South Asian ancestry. This finding aligns with previous genetic studies, which suggested a proto-Roma origin in the Indian subcontinent, followed by extensive gene flow during their diaspora, arrival in the Balkans, and subsequent dispersion across Europe. However, this genetic profile varies among Roma groups, showing a strong correlation between genetic ancestries and geographic location. Our results show that Western Roma (i.e. Iberian Roma) exhibit a significantly higher southwestern European component and lower southeastern European and South Asian components compared to Central and Eastern Roma groups. This patterns aligns with the long history of assimilation experienced by Roma in the Iberian Peninsula, where centuries of enforced settlement policies and assimilation laws likely led to greater incorporation of Iberian ancestry. These policies began with the 1499 *Pragmática* (a royal decree), which ordered Roma to abandon their nomadic lifestyle, acquire a trade, and serve a local lord, and culminated in the 1783 *Pragmática*, which granted citizenship but aimed at complete assimilation by prohibiting Roma culture and traditions (Martínez Dhier 2007; Sánchez 2022).

In contrast, the fragmented socio-political landscape of Central and Southeastern Europe contributed to greater endogamy and genetic isolation. Under the Ottoman Empire, Roma faced marginalisation but were generally tolerated, and settlement was not actively enforced (Marushiakova et al. 2001; Çelik 2004; Marsch 2008). However, extreme marginalisation persisted in the Ottoman suzerainties of Wallachia and Moldavia, where many Roma were segregated or enslaved (Gheorghe 1983; Achim 2004; Crowe 2016). In the Habsburg Empire, various assimilation laws were enacted, including four issued by Empress Maria Theresa (1758–1773) and a decree by Joseph II in 1783 (Crowe 2006; Zahra 2017). Despite strict sanctions, these measures were only partially effective, achieving lasting settlement in a few areas where Roma communities remain to this day, such as Burgenland in Austria (Klamper 1993; Halwachs 2005).

These differing historical circumstances likely shaped the genetic landscape of Roma populations, with greater isolation and endogamy in Eastern Roma groups leading to the preservation of a higher proportion of South Asian ancestry. This supports the notion that as Roma groups migrated westward, gene flow with local populations gradually increased. This differential gene flow may explain the distinct genetic profiles observed among European Roma groups, as demonstrated by our fineSTRUCTURE analysis, which shows that Iberian Roma form a separate cluster, while the rest of the European Roma exhibit a geographical structure. Specifically, the Czech and Macedonian Roma each form distinct clusters, while the remaining Roma samples are grouped together in a third cluster. The lack of substructure in some European Roma groups may be explained by the limited sample size.

The arrival of the Roma in Europe has been documented through historical and genetic data (Fraser 1992; Martínez-Cruz et al. 2016; Font-Porterias et al. 2019; Sánchez 2022). However, more recent migrations, such as mass movements that could be described as a second out-of-Balkans, have not been detected in previous genetic studies. Our admixture estimates performed with fastGLOBETROTTER and MALDER, suggest an earlier arrival of the proto-Roma to Eastern Europe (i.e., the European Roma groups analysed in our study) approximately 30–35 generations ago, compared to Western Europe (i.e., Iberian Roma) with estimates around 25–26 generations ago. In both cases, a significant northern Indian component is present in the admixture profiles, consistent with a North-western Indian origin of the proto-Roma (Fraser 1992; Hancock 2006; Gómez-Carballa et al. 2013; Martínez-Cruz et al. 2016; Bánfai et al. 2019; Font-Porterias et al. 2019), while other more specific ancestry components are evident in eastern and western Roma. These inferred dates slightly predate the historical records and may indicate that the earliest reports were recorded shortly after the actual arrival of the Roma to Europe. A key finding of our analysis is the evidence for previously undetected recent admixture in the Roma. Our fastGLOBETROTTER results reveal recent admixture events in both eastern and western Roma, occurring approximately 10 and 5 generations ago, respectively. The similarity of the ancestry components involved suggests ongoing migrations and gene flow during the 17th and 19th centuries among the Roma across Europe.

We assessed the effects of evolving social norms that have historically influenced relationships between Roma and non-Roma, and how these dynamics have been reflected in the genome. These changes in social norms were evaluated through the use of local ancestry tracts of European ancestry and shared IBD segments, as the presence of segments of different lengths point out to changes in admixture and endogamy over time (Baharian et al. 2016; Harris et al. 2018; Castro e Silva et al. 2022). We analysed the size and number of European ancestry tracts within Roma groups, as well as within- and between-population IBD segments, to evaluate variations in endogamy (within population) and gene flow (between populations). Finally, we categorised the segments by length and linked them to key events in Roma history. Observing the distribution of European local ancestry tracts, Iberian Roma displayed higher levels of endogamy in the past, particularly around the time of the arrival in Europe and the Iberian Peninsula. Over time, they experienced increasing gene flow with non-Roma Europeans, especially after the end of slavery and during the second out-of-Balkans migration event (Marushiakova and Popov 2010; Crowe et al. 2016), when many Roma migrated westward in search of opportunities (Marushiakova-Popova and Popov 2018). This contrasts with Southeastern European Roma groups, where gene flow with non-Roma Europeans decreased over time. Moreover, the distribution of shared IBD segments revealed an increase in endogamy levels among Southeastern European Roma and a decrease among Iberian Roma, suggesting a shift in mating patterns where, in recent times, Iberian Roma faced higher gene flow with the non-Roma, while Southeastern European Roma grew more isolated. This can also be attributed to differences in social structures between Iberian and Southeastern European Roma. These findings diverge from previous results (Mendizabal et al. 2012), where Roma from Spain, Portugal and Lithuania — all originating from a first out-of-Balkans event — showed a high number of short non-Roma European local ancestry tracts. Conversely, Southeastern European Roma groups had more long tracts. These discrepancies can be attributed to the significantly higher resolution of the current study, which included over six times more SNPs and a larger sample size for groups such as Spanish and Czech Roma, allowing a more accurate estimation of the ancestry segments.

To better understand the Roma community and their social history, we examined demographic dynamics, finding that all Roma groups have lower *N*_*e*_ values than non-Roma reference groups, with Iberian Roma showing a higher *N*_*e*_ than the other Roma groups. This difference likely stems from higher admixture with non-Roma Europeans and lower levels of isolation, which is consistent with the presence of the many long European local ancestry tracts detected in previous analyses. Interestingly, the *N*_*e*_ curve shows consistently low values across all Roma groups over the past 50 generations. After hitting its lowest point between 7 and 29 generations ago, the average *N*_*e*_ of Roma groups began to rise, suggesting a reduction in endogamy due to migration and increased gene flow with other populations. This trend is supported by the inferred admixture events (arrival into Europe and arrival into the Iberian Peninsula), aligning with earlier research (Bianco et al. 2020).

Continuing our focus on social and cultural dynamics, we conducted a detailed analysis of endogamy patterns within Roma populations by examining the distribution of ROH and IBD segments. Consanguineous unions increase the likelihood of identical genomic segments being paired within individuals, leading to the formation of ROHs (Severson et al. 2019). The presence of both short and long ROHs within Roma indicates historically high levels of inbreeding (understood as mating between relatives, (Ceballos et al. 2018), consistent with previous research (Mendizabal et al. 2012; Bianco et al. 2020; Font-Porterias et al. 2021). This is further supported by examining IBD segments, where the abundance of shared segments of any length between Roma individuals confirms the presence of a historical pattern of isolation. Specifically, the high number of segments under 8 cM of length suggests that endogamy likely peaked in a period that goes from ~ 1000 to ~ 500 years ago, corresponding to the lowest historical *N*_*e*_ and the estimated older dates of admixture inferred by fastGLOBETROTTER.

Assortative mating is a form of sexual selection that shapes the characteristics and reproduction of communities. Genetic evidence now shows for the first time that Roma are subject to this process, as evidenced by ANCESTOR results showing that Roma tend to choose partners with similar ancestry proportions. With their distinctive ancestry profiles, this means that Roma partners are often chosen from within the same community, which contributes to higher levels of endogamy. This mating pattern is reflected in the positive correlation between European and South-Asian ancestries across all groups. These findings align with cultural studies (Weyrauch 2001; Drummond 2011; Gamella and Álvarez-Roldán 2023) and highlight the significant role of non-random mating on the genetic profile of Roma communities.

The availability of extensive data from Iberian individuals enables a detailed analysis of the Roma’s microgeographical genomic structure. Our findings reveal a distinct ancestry profile and genetic substructure between Iberian Roma and non-Roma individuals. The AMOVA indicates that Iberian Roma groups exhibit a level of heterogeneity and geographical structure ten times greater than that of non-Roma groups. Despite this genetic substructure, the allele-sharing between Iberian Roma and non-Roma groups does not demonstrate significant within-region genetic similarity. For example, Roma from Eastern Iberia do not exhibit more allele-sharing with non-Roma from the same region than with other non-Roma Iberians, suggesting that inter-group mating has occurred randomly without a clear regional pattern.

We also explored the presence of alternative ancestries with the Iberian Roma, considering their cohabitation with Christians, Jews, and Muslim North Africans from their arrival on the Iberian Peninsula in the 15th century until the expulsion of Jews and Muslim North Africans (Pérez and Hochroth 2007; Carr 2017). F-statistics indicate that non-Roma groups are genetically closer to Jews and North Africans than to the Roma, with the sole exception of Cochin Jews (from southern India), who share closer ties with the Roma due to their Indian ancestry. A small fraction of North African ancestry is present in both Iberian Roma (2–3%) and non-Roma (11%) but is absent in other Roma groups, suggesting that this North African component in Iberian Roma likely results from admixture with Iberian non-Roma after their arrival on the Peninsula. Similarly, the Jewish ancestry found in Iberian Roma (1%) likely entered their gene pool through admixture with Iberian populations (being 2% in IBS; Supplementary Fig. 26a-b) after their arrival. These results indicate minimal historical interaction between Roma, Muslims, and Jews in the Iberian Peninsula, challenging hypotheses of Jewish descent or North African migration in Roma origins (Aparicio Gervás 2006; Hancock 2006; Pohoryles 2018). Our findings support a South Asian origin for the Roma, followed by a diaspora across western Asia before reaching the Balkans and eventually dispersing across Europe until their arrival on the Iberian Peninsula, consistent with previous studies (Mendizabal et al. 2011; Martínez-Cruz et al. 2016; Bianco et al. 2020).

This study is the first to provide a genetic characterisation of the Roma, confirming their Indian origins, Central European migration routes, and revealing a genetic substructure marked by varying levels of non-Roma admixture, shaped by complex historical events and evolving social norms. Despite challenges such as uneven sample sizes and the absence of key reference groups, our findings suggest that the Roma are a highly admixed and evolving community, shaped by centuries of migrations and interactions with other populations, and highlight the need for further research, including whole-genome sequencing and studies of underrepresented Roma communities.

## Materials and methods

### Sampling, dataset assembly, and quality control

The present study is based on genome-wide data of 105 self-reported Spanish Roma, 5 Portuguese Roma (Font-Porterias et al. 2019, 2021), and 42 Roma individuals sampled in the Czech Republic (see Data Availability), all genotyped using the Affymetrix Axiom Genome-Wide Human Origins 1 array, which includes 630k genome-wide SNPs. Additionally, 52 Czech non-Roma controls were genotyped with the same array for this study. Beyond the newly generated array data, we included previously published sequence data from 29 Roma individuals from other European regions (North Macedonia, Lithuania, Hungary, Ukraine) from Bianco et al. 2020. As the Roma populations exhibit appreciable population substructure, the inclusion of Czech and Eastern European Roma alongside Iberian Roma aimed to enhance the representation of Roma genetic diversity across Europe, allowing for a comparative analysis of genetic differentiation and admixture patterns between Western and Central European Roma groups.

For the genotyped Czech samples and the complete genomes in the Bianco et al. (Bianco et al. 2020) dataset, the variant calling using GATK (McKenna et al. 2010) was performed following the GATK Best Practices pipeline (Auwera et al. 2013). Sequencing reads were aligned to the GRCh37 human reference genome using BWA-MEM v 0.7.12 (Li 2013), removing PCR duplicates with Picard Tools v 2.18.6 (Picard Toolkit 2019), and performing base quality recalibration using GATK’s BaseRecalibrator (BQSR). We performed joint genotype calling (HaplotypeCaller and GenotypeGVCFs), and we used GATK DepthOfCoverage program (--minBaseQuality 20 --minMappingQuality 20) to estimate sequencing depth. The final call set consists of 613,535 autosomal SNPs in the Czech dataset and 28,678,694 autosomal SNPs in the Bianco dataset.

Besides the Roma samples described above, reference samples from previously published whole genome sequences and genome-wide data from Europe, Caucasus, Central Africa, Central and Southern Asia (Lazaridis et al. 2014; Auton et al. 2015), as well as North Africa (Patterson et al. 2012; Lucas-Sánchez et al. 2023), were included in the analyses. Following the approach used in Font-Porterias et al. (2019), populations from the reference dataset were standardised to include 25 individuals to mitigate potential biases caused by highly imbalanced sample sizes while maintaining optimal representation. However, the non-Roma Spanish population (IBS) and Punjabi from Lahore (PJL) from the 1000 Genomes Project were excluded from this standardisation due to their pivotal relevance in the analysis of Roma. Using PLINK 2 (Chang et al. 2015), SNPs with a missingness rate higher than 5% and with a minor allele frequency below 0.05 were removed, and individuals with more than 10% of missing calls were removed. We used vcftools (Danecek et al. 2011) to extract biallelic SNPs and to check for related individuals using the included relatedness2 method; we then removed individuals related above the third degree of relatedness (values of kinship coefficient between 0.0442 and 0.5). The final dataset includes 335,367 autosomal SNPs in 1,189 individuals after filtering for QC (Supplementary Table 13), including 95 Iberian Roma and 21 Czech Roma unrelated individuals.

In our analyses, we refer to the combined Spanish and Portuguese Roma samples as the Iberian Roma. For some specific analyses we selected a subset of individuals containing exclusively Iberian Roma and IBS populations clustered by geographic regions (henceforth called Iberian dataset). The Iberian Roma were classified into five geographical regions (Centre, North, South, West, and East; Supplementary Fig. 27) based on their parents and grandparents’ birthplaces, similar to the approach used in Aizpurua-Iraola et al. (Aizpurua-Iraola et al. 2022). In the cases where no information about the geographical origin of the volunteer and volunteer ancestors was available, or the cases where ancestors originated in different geographical areas, they were classified as *‘Indeterminate’*. For the *IBS* population from the 1000GP dataset our classification strategy consisted in assigning each individual to the respective region of their sampling location. Due to the lack of detailed information about birthplace or family origins for the Roma from Central and Eastern Europe, they were assigned to their respective countries based solely on the sampling location.

(distribution of the Roma samples in Fig. 1a).

### Principal component analysis (PCA) and population structure

Data was pruned for linkage disequilibrium using PLINK v2, removing SNPs with an r² > 0.5 in a sliding window of 200 SNPs, and steps of 25 SNPs. The pruned dataset contains 156,153 SNPs, which were then used to perform a PCA with SmartPCA from the EIG v6.0.1 software package (Price et al. 2006; Patterson et al. 2006) on the complete and the Iberian datasets.

To infer the global ancestry components in the Roma populations, ADMIXTURE v1.3.0 (Alexander and Lange 2011) was used with 10 iterations of cross-validation error calculation and they were plotted using PONG v1.4.9.

To estimate within-population and between-population genetic variation, an analysis of the molecular variance (AMOVA) was conducted, and diversity indices were calculated using the Poppr R package (Kamvar et al. 2014; Behr et al. 2016) on the Iberian dataset.

### Patterns of allele sharing

AdmixTools2 (Maier et al. 2023) was used to estimate allele-sharing between the Roma populations and putative parental source populations from Europe, Asia, and Africa. To assess evidence of admixture in Roma populations, we conducted *f3* tests in the form of *f3*(Roma; Source1, Source2). To investigate the shared drift between the Roma and other populations from a common outgroup, we employed the three-population test (*f3*) using the entire dataset. This was done through the outgroup *f3*-statistic: *f3* (YRI; Roma, X), where YRI represents Yoruba individuals from the 1000GP (Auton et al. 2015), Roma represents any Roma population in the dataset, and X denotes any other population.

To infer more specific patterns of allele-sharing, *f4* statistics were computed in the following way: (i) *f4*(Yoruba; Roma, Source1; Source2). The source populations used in the tests were selected as follows: (i) pairs of European reference populations; (ii) pairs of Asian reference populations; and (iii) pairs of North African and Jewish reference populations. In addition, we estimated *f4*-statistics using the Iberian dataset, to infer if patterns of allele-sharing were geographically-related between Roma and non-Roma from the Iberian Peninsula, in the way *f4*(Yoruba; Roma, IBS; IBS). To avoid biases, in the *f3* outgroup test on the complete dataset and the *f4* tests with European references only the 25 IBS individuals with the lowest North African ancestry component were used, measured according to a Local Ancestry Inference (LAI) (Supplementary Table 14).

### Phasing

The haplotype phase was inferred for each chromosome using default settings with SHAPEIT v2 (Delaneau et al. 2013), using the HapMap GRCh37 genetic map (Gibbs et al. 2003) and 1000 Genomes (Phase 3) as a reference panel (Auton et al. 2015). In a pre-phasing step carried out (--check) to remove any SNPs that did not align correctly to the reference no SNP was removed. Specifically, for the identity-by-descent (IBD) analyses and IBD-based estimation of effective population size, we phased autosomal data using Beagle v 5.3 (Browning et al. 2021), following the pipeline proposed by Browning et al. (Browning et al. 2018).

### Haplotype-sharing analysis

To detect fine-scale population structure, we performed ChromoPainter/fineSTRUCTURE analysis (Lawson et al. 2012; Hellenthal et al. 2014) on the complete dataset, first with the Romani populations as target, then with the *IBS* population as target, to provide a baseline of comparison for the results. We used the ChromoPainter v2 (Lawson et al. 2012) expectation-maximisation (EM) algorithm to obtain the global mutation probability (M) and the switch rate (n) parameters by setting the -in -iM switches. This analysis was performed on chromosomes 1, 7, 14, and 20, with 15 iterations of the EM algorithm, and the -a switch was used to parallelise the analysis for each subset of 20 individuals, from a total of 1,189. The resulting parameters were averaged across chromosomes, weighted by the number of SNPs per chromosome, to obtain the final values of *n* = 252.38 and M = 0.0006. Next, we used ChromoPainter with the previously estimated parameters, to infer number and length of shared haplotypes between every pair of individuals in the dataset, producing the coancestry matrix. ChromoCombine was then used to combine the results over all the chromosomes.

FineSTRUCTURE v4.1.1 (Hellenthal et al. 2014) was used on the coancestry matrix to group the data and determine genetic clusters based on patterns of haplotype sharing. The analysis was conducted for 2 million iterations using Markov Chain Monte Carlo, with 1 million iterations designated as “burn-in,” and sampling of values was performed every 10,000 iterations. For the complete dataset analysis, FineSTRUCTURE was run with the normalisation parameter “c” estimated as 0.262. FineSTRUCTURE dendrograms were built with the default parameter -m T.

The analysis was performed for three different seeds in the chunkcounts and chunklengths sharing coancestry matrices from ChromoPainter, and the consistency of the dendrograms between different seeds was manually evaluated. The three chunkcounts dendrograms were used as references to assign individuals to genetic clusters.

Following that, a third ChromoPainter analysis was run (Donors vs. Target), setting the -f switch to state which individuals belong to each cluster and which genetic clusters would be donor groups. In the population file, all the clusters were set both as donors (D) and recipients (R), except for the Roma clusters, which were only set as recipients. A modified version of the NNLS method implemented in the *nnls* v1.4 R package (Katharine M. Mullen and Ivo H. M. van Stokkum 2012) was used to infer the ancestry profiles of the clusters using the ChromoPainter coancestry matrix. The Donor clusters were combined into macroregions defined according to geographical criteria (i.e. European, Middle Eastern) for ease of representation.

Using the NNLS ancestry component percentages, Pearson’s correlation tests were performed to explore the potential presence of a geographic cline of variation in the ancestral components of Roma individuals (see Supplementary Note 3).

### Dating admixture events

The fastGLOBETROTTER method (Wangkumhang et al. 2022) was used to infer and date admixture events in our sample sets. For our analysis, we ran fastGLOBETROTTER to detect admixture events in Europe and in the Iberian Peninsula. All the donor clusters were used as surrogate populations to represent the admixing sources in the analyses. We applied ChromoPainter v2 to paint Roma individuals using the surrogate population only as donors and inferred copying vectors for the target individuals.

fastGLOBETROTTER was run for 5 mixing iterations using the prop.ind:1 and null.ind:1 settings. After reviewing the results and evaluating clusters with multiple or single-date admixture, we conducted an additional run with fastGLOBETROTTER using 100 bootstrap resampling iterations to estimate the admixture dates, following the developer’s approach for measuring 95% confidence intervals (CI) around inferred dates (Wangkumhang et al. 2022). The mean admixture date was calculated as the average across bootstrap estimates for each event, with CIs and Standard Errors derived from the bootstraps obtained using the standard model (null.ind: 1). A generation time of 25 years was assumed, as done in previous comparable studies (Martínez-Cruz et al. 2016; Font-Porterias et al. 2019; Vilà-Valls et al. 2023). The sources of each admixture event were combined into the previously defined macro-regions for ease of visualisation.

As an alternative estimate of the admixture events, MALDER (Pickrell et al. 2014) was run on the non-pruned and unphased dataset containing individuals from the IberianRoma and EuropeanRoma clusters. We used a set of reference clusters that reflected the diaspora route, with the addition of Basque for the IberianRoma test. The dataset was first converted from plink to Eigenstrat format using the *convertf* function from EIG v 6.0.1 (Patterson et al. 2006). Then, MALDER was run with a minimum distance of 0.005 cM and jackknife resampling. Similar to the fastGLOBETROTTER results, we converted time from admixture in generations to a date by assuming a generation time of 25 years.

### Homozygosity and identity-by-descent fragment Estimation

Runs of homozygosity (ROH) were identified using PLINK v2, considering runs with at least 50 SNPs, a minimum length of 500 kb, and a maximum gap of 100 kb between two consecutive SNPs. The ROH lengths were categorised into four length categories: very short (< 1 Mb), short (1 to 2.5 Mb), medium (2.5 to 5 Mb), and long (> 5 Mb).

Using the dataset phased with Beagle, we identified IBD segments between pairs of individuals. To call IBD blocks, we used Hap-Ibd (Zhou et al. 2020) on the main dataset, utilising the HapMap GRCh37 genetic map to convert from base pairs to genetic positions in centiMorgans (cM). We excluded any segment under 3 cM in length to limit overestimations. After the estimation, we merged the segments using the provided tool (merge-ibd-segments.17Jan20.102.jar) from the Browning et al. (Browning et al. 2018) pipeline. To construct an IBD heatmap, we summed the IBD pairwise lengths between individuals following Han et al. (Han et al. 2017) and used the heatmaply package to plot the results in R (Galili et al. 2018). To explore variations in endogamy and gene flow between populations over time, we assessed the within- and between-population IBD sharing patterns in Roma and non-Roma European populations (see Supplementary Note 1).

### Effective population size (*N*e) Estimation

We further explored the demographic history of the Roma population using the measured IBD segments to estimate historical changes in effective population size (Ne) using IBDNe (Browning and Browning 2015). The analysis utilised the HapMap GRCh37 genetic map as a reference. We set a minimum threshold to exclude segments of IBD shorter than 3 cM. To generate a 95% CI for the results, we employed a bootstrap approach with 100 simulations.

Initially, the analysis was performed on all Roma groups and then on the IBS, Czech, and PJL reference populations. As this method is most reliable for recent periods when applied to genome-wide array data (Browning and Browning 2015), we filtered the data to retain only information from the present up to 50 generations ago. The 50-generation cutoff was chosen based on Browning and Browning (2015), which demonstrated that SNP array data provides reliable estimates of effective population size within this timeframe. Beyond 50 generations, increased uncertainty in IBD-segment detection leads to underestimation of effective population size, making the method less accurate.

### Tests for ancestry-assortative mating

We conducted tests for ancestry-assortative mating using ANCESTOR. ANCESTOR is an algorithm that leverages phased local ancestry tracts to estimate the ancestral proportions of both parents for each individual. A RFMix 2 (Maples et al. 2013; Zou et al. 2015a, b) analysis was performed to generate the LAI used by ANCESTOR, using a two-group reference set consisting of European and South-Asian populations. To convert RFMix output to the required inputs we used the code from Korunes et al. (Korunes et al. 2022). The European reference group comprised 95 individuals from IBS and Balkan populations (Croatian, Greek, Hungarian, Romanian), while the South-Asian group was formed by 95 Punjabi (PJL) individuals. Using the inferred parental ancestries, we tested for assortative mating, which is indicated by a positive correlation in ancestry between the inferred mating pairs.

### Local ancestry tracts size distribution

We aimed to infer the ancestry tract size distribution to compare the observed patterns for various Roma populations, with the goal of determining which groups exhibit early and later admixture with non-Roma European populations (or, at least, when this admixture was most intense). To do this, first the local ancestry inference was performed using RFMix v2 software, using two reference populations, European and South-Asian (see Supplementary Note 4). Then we identified the ancestry tracts and their length, using an in-house developed tool (details in Supplementary Note 2). Lastly, the length tract distributions of different Roma populations were compared, pooling all tracts together and then splitting them in length size categories.

## Electronic supplementary material

Below is the link to the electronic supplementary material.

Supplementary Material 1

Supplementary Material 2

## Data Availability

The genomic data of Czech Roma and non-Roma individuals analysed during the current study is available in the EGA repository, under accession number EGAD50000001103. The scripts generated during this study can be downloaded from https://github.com/gfena/AncestryLength

## References

[CR1] Abreu L (2007) Beggars, vagrants and romanies: repression and persecution in Portuguese society (14th–18th Centuries). Hygiea 6:41–66. 10.3384/hygiea.1403-8668.076141

[CR2] Achim V (2004) The Roma in Romanian history. Central European University

[CR3] Aizpurua-Iraola J, Giménez A, Carballo-Mesa A et al (2022) Founder lineages in the Iberian Roma mitogenomes recapitulate the Roma diaspora and show the effects of demographic bottlenecks. Sci Rep 12:18720. 10.1038/s41598-022-23349-9 36333436 10.1038/s41598-022-23349-9 PMC9636147

[CR4] Alexander DH, Lange K (2011) Enhancements to the ADMIXTURE algorithm for individual ancestry Estimation. BMC Bioinformatics 12:246. 10.1186/1471-2105-12-246 21682921 10.1186/1471-2105-12-246 PMC3146885

[CR5] Aparicio Gervás JM (2006) Breve recopilación sobre La historia Del Pueblo Gitano: desde Su Salida Del Punjab, Hasta La constitución Española de 1978. Veinte Hitos sobre La Otra historia de España. RIFOP: revista interuniversitaria de Formación Del Profesorado: continuación de La Antigua. Revista De Escuelas Normales 141–162

[CR6] Auton A, Abecasis GR, Altshuler DM et al (2015) A global reference for human genetic variation. Nat 2015 526:7571. 10.1038/nature15393 10.1038/nature15393 PMC4750478 26432245

[CR8] Baharian S, Barakatt M, Gignoux CR et al (2016) The great migration and African-American genomic diversity. PLoS Genet 12:e1006059. 10.1371/journal.pgen.1006059 27232753 10.1371/journal.pgen.1006059 PMC4883799

[CR9] Bánfai Z, Melegh BI, Sümegi K et al (2019) Revealing the genetic impact of the Ottoman occupation on ethnic groups of East-Central Europe and on the Roma population of the area. Front Genet 10:558 31263480 10.3389/fgene.2019.00558 PMC6585392

[CR10] Behr AA, Liu KZ, Liu-Fang G et al (2016) Pong: fast analysis and visualization of latent clusters in population genetic data. Bioinformatics 32:2817–2823. 10.1093/bioinformatics/btw327 27283948 10.1093/bioinformatics/btw327 PMC5018373

[CR11] Bernát A, Messing V (2016) Methodological and data infrastructure report on Roma population in the EU. In:GRID

[CR12] Bianco E, Laval G, Font-Porterias N et al (2020) Recent Common Origin, Reduced Population Size, and Marked Admixture Have Shaped European Roma Genomes. Molecular Biology and Evolution. 10.1093/molbev/msaa156 10.1093/molbev/msaa156 32589725

[CR13] Bover O, Velilla P (1999) Migration in Spain. Historical Background and Current Trends

[CR14] Brearley M (2001) The persecution of Gypsies in Europe. Am Behav Sci 45:588–599. 10.1177/00027640121957367

[CR16] Browning SR, Browning BL (2015) Accurate Non-parametric Estimation of recent effective population size from segments of identity by descent. Am J Hum Genet 97:404–418. 10.1016/j.ajhg.2015.07.012 26299365 10.1016/j.ajhg.2015.07.012 PMC4564943

[CR17] Browning SR, Browning BL, Daviglus ML et al (2018) Ancestry-specific recent effective population size in the Americas. PLoS Genet 14:e1007385. 10.1371/journal.pgen.1007385 29795556 10.1371/journal.pgen.1007385 PMC5967706

[CR15] Browning BL, Tian X, Zhou Y, Browning SR (2021) Fast two-stage phasing of large-scale sequence data. Am J Hum Genet 108:1880–1890. 10.1016/j.ajhg.2021.08.005 34478634 10.1016/j.ajhg.2021.08.005 PMC8551421

[CR18] Campbell CL, Palamara PF, Dubrovsky M et al (2012) North African Jewish and non-Jewish populations form distinctive, orthogonal clusters. Proceedings of the National Academy of Sciences 109:13865–13870. 10.1073/pnas.1204840109 10.1073/pnas.1204840109 PMC3427049 22869716

[CR19] Carr M (2017) Blood and faith: the purging of Muslim Spain. Oxford University Press, pp 1492–1614

[CR20] Castro e Silva MA, Ferraz T, Couto-Silva CM et al (2022) Population histories and genomic diversity of South American natives. Mol Biol Evol 39:msab339. 10.1093/molbev/msab339 34875092 10.1093/molbev/msab339 PMC8789086

[CR21] Ceballos FC, Joshi PK, Clark DW et al (2018) Runs of homozygosity: windows into population history and trait architecture. Nat Rev Genet 19:220–234. 10.1038/nrg.2017.109 29335644 10.1038/nrg.2017.109

[CR22] Çelik F (2004) Exploring marginality in the Ottoman Empire: Gypsies or People of Malice (Ehl-i Fesad) as viewed by the Ottomans. EUI WORKING PAPERS:RSCAS No. 2004/39

[CR23] Chang CC, Chow CC, Tellier LC et al (2015) Second-generation PLINK: rising to the challenge of larger and richer datasets. GigaScience 4:s13742-015-0047–8. 10.1186/s13742-015-0047-8 10.1186/s13742-015-0047-8 PMC4342193 25722852

[CR26] Crowe DM (2006) From persecution to pragmatism: the Habsburg Roma in the eighteenth century. Austrian History Yearbook 37:99–120

[CR24] Crowe D (2016) The Gypsy historical experience in Romania. The Gypsies of Eastern Europe. Routledge, pp 61–79

[CR25] Crowe D, Kolsti J, Hancock I (2016) The Gypsies of Eastern Europe. Routledge

[CR27] Danecek P, Auton A, Abecasis G et al (2011) The variant call format and vcftools. Bioinformatics 27:2156–2158. 10.1093/bioinformatics/btr330 21653522 10.1093/bioinformatics/btr330 PMC3137218

[CR28] Delaneau O, Zagury J-F, Marchini J (2013) Improved whole-chromosome phasing for disease and population genetic studies. Nat Methods 10:5–6. 10.1038/nmeth.2307 23269371 10.1038/nmeth.2307

[CR7] der Auwera GAV, Carneiro MO, Hartl C et al (2013) From FastQ data to high-confidence variant calls: the genome analysis toolkit best practices pipeline. Curr Protocols Bioinf 43. 10.1002/0471250953.bi1110s43 10.1002/0471250953.bi1110s43 PMC4243306 25431634

[CR29] Drummond S (2011) Mapping marriage law in Spanish Gitano communities. UBC

[CR30] Ena GF, Aizpurua-Iraola J, Font-Porterias N et al (2022) Population genetics of the European Roma—A. Rev Genes 13:2068. 10.3390/genes13112068 10.3390/genes13112068 PMC9690732 36360305

[CR31] Font-Porterias N, Arauna LR, Poveda A et al (2019) European Roma groups show complex West Eurasian admixture footprints and a common South Asian genetic origin. PLoS Genet 15:e1008417. 10.1371/journal.pgen.1008417 31545809 10.1371/journal.pgen.1008417 PMC6779411

[CR32] Font-Porterias N, Giménez A, Carballo-Mesa A et al (2021) Admixture has shaped Romani genetic diversity in clinically relevant variants. Front Genet 12:683880. 10.3389/fgene.2021.683880 34220960 10.3389/fgene.2021.683880 PMC8244592

[CR33] Fraser AM (1992) The Gypsies (The peoples of Europe). Blackwell Pub

[CR34] Galili T, O’Callaghan A, Sidi J, Sievert C (2018) Heatmaply: an R package for creating interactive cluster heatmaps for online publishing. Bioinformatics 34:1600–1602. 10.1093/bioinformatics/btx657 29069305 10.1093/bioinformatics/btx657 PMC5925766

[CR35] Gamella JF, Álvarez-Roldán A (2023) Breaking secular endogamy. The growth of intermarriage among the Gitanos/Calé of Spain (1900–2006). History Family 28:457–483

[CR36] García-Fernández C, Font-Porterias N, Kučinskas V et al (2020) Sex-biased patterns shaped the genetic history of Roma. Sci Rep 10:14464. 10.1038/s41598-020-71066-y 32879340 10.1038/s41598-020-71066-y PMC7468237

[CR37] General Assembly of the World Medical Association (2014) World medical association declaration of Helsinki: ethical principles for medical research involving human subjects. J Am Coll Dent 81:14–18 25951678

[CR38] Gheorghe N (1983) The origin of Roma’s slavery in the Romanian principalities. Roma 7:12–27

[CR39] Gibbs RA, Belmont JW, Hardenbol P et al (2003) The international HapMap project. Nature. 10.1038/nature02168

[CR113] Giménez A, Comas D, Carballo A (2019) Origen e identidad del Pueblo Gitano. Int J Roma Stud 1:159–184 10.17583/ijrs.2019.4561

[CR40] Gómez-Carballa A, Pardo-Seco J, Fachal L et al (2013) Indian signatures in the Westernmost edge of the European Romani diaspora: new insight from mitogenomes. PLoS ONE 8:e75397. 10.1371/journal.pone.0075397 24143169 10.1371/journal.pone.0075397 PMC3797067

[CR41] Greenberg J (2010) Report on Roma education today: from slavery to segregation and beyond. Colum L Rev 110:919–1001

[CR42] Gusmão A, Gusmão L, Gomes V et al (2008) A perspective on the history of the Iberian Gypsies provided by phylogeographic analysis of Y-Chromosome lineages. Ann Hum Genet 72:215–227. 10.1111/j.1469-1809.2007.00421.x 18205888 10.1111/j.1469-1809.2007.00421.x

[CR43] Halwachs D (2005) Roma and Romani in Austria. Romani Stud 15:145–173

[CR44] Han E, Carbonetto P, Curtis RE et al (2017) Clustering of 770,000 genomes reveals post-colonial population structure of North America. Nat Commun 8:14238 28169989 10.1038/ncomms14238 PMC5309710

[CR45] Hancock I (2006) On Romani origins and identity. The Romani Archives and Documentation Center, Aralık

[CR46] Harris DN, Song W, Shetty AC et al (2018) Evolutionary genomic dynamics of Peruvians before, during, and after the Inca Empire. Proceedings of the National Academy of Sciences 115:E6526–E6535. 10.1073/pnas.1720798115 10.1073/pnas.1720798115 PMC6048481 29946025

[CR47] Hellenthal G, Busby GBJ, Band G et al (2014) A genetic atlas of human admixture history. Science 343:747–751. 10.1126/science.1243518 24531965 10.1126/science.1243518 PMC4209567

[CR48] Henn BM, Botigué LR, Gravel S et al (2012) Genomic ancestry of North Africans supports Back-to-Africa migrations. PLoS Genet 8:e1002397. 10.1371/journal.pgen.1002397 22253600 10.1371/journal.pgen.1002397 PMC3257290

[CR49] Iovita RP, Schurr TG (2004) Reconstructing the origins and migrations of diasporic populations: the case of the European Gypsies. Am Anthropol 106(2):267–281

[CR50] Ivanov A, Kagin J (2014) Roma poverty from a human development perspective. Istanbul, Turkey, UNDP, p 96

[CR51] Kajanova A, Kmecova I (2018) Development of unemployment of the Roma minority in selected European countries. Cswhi 9:59–64. 10.22359/Cswhi_9_4_08

[CR52] Kalaydjieva L, Gresham D, Calafell F (2001) Genetic studies of the Roma (Gypsies): a review. BMC Med Genet 2:5. 10.1186/1471-2350-2-5 11299048 10.1186/1471-2350-2-5 PMC31389

[CR53] Kamvar ZN, Tabima JF, Grünwald NJ (2014) Poppr: an R package for genetic analysis of populations with clonal, partially clonal, and/or sexual reproduction. PeerJ 2:e281. 10.7717/peerj.281 24688859 10.7717/peerj.281 PMC3961149

[CR54] Katharine M, Mullen, Ivo HM, van Stokkum (2012) The Lawson-Hanson algorithm for non-negative least squares (NNLS). https://citeseerx.ist.psu.edu/document?repid=rep1%26type=pdf%26doi=b84fcc0feb78f074edad012273847ce8fa5361ce. Accessed 1 Jul 2024

[CR55] Kenrick D (2007) Historical dictionary of the Gypsies (Romanies). Scarecrow

[CR56] Kenrick D, Puxon G (2009) Gypsies under the swastika. University of Hertfordshire

[CR57] Kimmel S (2015) Parables of coercion: conversion and knowledge at the end of Islamic Spain. University of Chicago Press

[CR58] Klamper E (1993) Persecution and annihilation of Roma and Sinti in Austria, 1938–1945. Romani Stud 3:55

[CR59] Korunes K, KL, Soares-Souza GB, Bobrek et al (2022) Sex-biased admixture and assortative mating shape genetic variation and influence demographic inference in admixed Cabo Verdeans. G3 Genes|Genomes|Genetics 12:jkac183. 10.1093/g3journal/jkac183 10.1093/g3journal/jkac183 PMC9526050 35861404

[CR60] La Parra-Casado D, Mosquera PA, Vives-Cases C, San Sebastian M (2018) Socioeconomic inequalities in the use of healthcare services: comparison between the Roma and general populations in Spain. Int J Environ Res Public Health 15:121 29329246 10.3390/ijerph15010121 PMC5800220

[CR61] Laparra M (2007) Informe sobre La situación social y tendencias de Cambio En La Población gitana. Ministerio de Trabajo y Asuntos Sociales, Una primera aproximación Madrid

[CR62] Laparra M, Arza J, Fernández A (2007) Diagnóstico social de La Comunidad gitana En España, vol 2011. Un análisis contrastado de la Encuesta del CIS a Hogares de Población Gitana

[CR63] Lawson DJ, Hellenthal G, Myers S, Falush D (2012) Inference of population structure using dense haplotype data. PLoS Genet 8:e1002453. 10.1371/journal.pgen.1002453 22291602 10.1371/journal.pgen.1002453 PMC3266881

[CR64] Lazaridis I, Patterson N, Mittnik A et al (2014) Ancient human genomes suggest three ancestral populations for present-day Europeans. Nature 513:409–413. 10.1038/nature13673 25230663 10.1038/nature13673 PMC4170574

[CR65] Leblon B (1985) Les Gitans d’Espagne: Le prix de la différence. FeniXX

[CR66] Lewy G (2000) The Nazi persecution of the Gypsies. Oxford University Press

[CR67] Li H (2013) Aligning sequence Reads, clone. sequences and assembly contigs with BWA-MEM

[CR68] Lucas-Sánchez M, Fadhlaoui-Zid K, Comas D (2023) The genomic analysis of current-day North African populations reveals the existence of trans-Saharan migrations with different origins and dates. Hum Genet 142:305–320. 10.1007/s00439-022-02503-3 36441222 10.1007/s00439-022-02503-3 PMC9918576

[CR69] Maier R, Flegontov P, Flegontova O et al (2023) On the limits of fitting complex models of population history to f-statistics. eLife 12:e85492. 10.7554/eLife.85492 37057893 10.7554/eLife.85492 PMC10310323

[CR70] Maples BK, Gravel S, Kenny EE, Bustamante CD (2013) RFMix: A discriminative modeling approach for rapid and robust Local-Ancestry inference. Am J Hum Genet 93:278–288. 10.1016/j.ajhg.2013.06.020 23910464 10.1016/j.ajhg.2013.06.020 PMC3738819

[CR71] Martínez Dhier A (2007) La condición social y jurídica de los gitanos en la legislación histórica española.(A partir de la pragmática de los Reyes Católicos de 1499). Doctoral thesis, Universidad de Granada

[CR72] Martínez-Cruz B, Mendizabal I, Harmant C et al (2016) Origins, admixture and founder lineages in European Roma. Eur J Hum Genet 24:937–943. 10.1038/ejhg.2015.201 26374132 10.1038/ejhg.2015.201 PMC4867443

[CR73] Marushiakova E, Popov V (2001) Gypsies in the Ottoman empire: A contribution to the history of the Balkans, vol 22. University of Hertfordshire

[CR74] Marushiakova E, Popov V (2010) Gypsy/Roma European migrations from 15th century till nowadays. In: Proceedings of International Conference Romani Mobilities in Europe: Multidisciplinary Perspectives. pp 126–139

[CR75] Marushiakova-Popova EA, Popov V (2018) Migration vs. inclusion: Roma mobilities from East to West. Baltic Worlds

[CR76] Matras Y (2002) Romani A linguistic introduction. Cambridge University Press

[CR77] McKenna A, Hanna M, Banks E et al (2010) The genome analysis toolkit: A mapreduce framework for analyzing next-generation DNA sequencing data. Genome Res 20:1297. 10.1101/GR.107524.110 20644199 10.1101/gr.107524.110 PMC2928508

[CR78] Mendes MM, Magano O (2022) Roma/Ciganos and the condition of internal strange in Portuguese society: the construction of otherness. In: Simmel and beyond. Routledge

[CR80] Mendizabal I, Valente C, Gusmão A et al (2011) Reconstructing the Indian origin and dispersal of the European Roma: A maternal genetic perspective. PLoS ONE 6:1–10. 10.1371/journal.pone.0015988 10.1371/journal.pone.0015988 PMC3018485 21264345

[CR79] Mendizabal I, Lao O, Marigorta UM et al (2012) Report reconstructing the population history of European Romani from Genome-wide data. Curr Biol 22:2342–2349. 10.1016/j.cub.2012.10.039 23219723 10.1016/j.cub.2012.10.039

[CR81] Moorjani P, Patterson N, Loh P-R et al (2013) Reconstructing Roma history from Genome-Wide data. 10.1371/journal.pone.0058633. PLOS One 8:10.1371/journal.pone.0058633 PMC3596272 23516520

[CR83] O’Nions H (2011) Roma expulsions and discrimination: the elephant in Brussels. Eur J Migration Law 13:361–388. 10.1163/157181611X605864

[CR82] O’Nions H (2016) Minority rights protection in international law: the Roma of Europe. Routledge, London

[CR84] Ortega MHS (1994) Los Gitanos Españoles desde Su Salida de La India Hasta Los Primeros conflictos En La Península. Espacio Tiempo y Forma Serie IV, Historia Moderna

[CR85] Pamjav H, Zalán A, Béres J et al (2011) Genetic structure of the paternal lineage of the Roma people. Am J Phys Anthropol 145:21–29. 10.1002/ajpa.21454 21484758 10.1002/ajpa.21454

[CR86] Parekh N, Rose T (2011) Health inequalities of the Roma in Europe: a literature review. Cent Eur J Public Health 19:139–142. 10.21101/cejph.a3661 22026288 10.21101/cejph.a3661

[CR87] Patterson N, Price AL, Reich D (2006) Population structure and eigenanalysis. PLoS Genet 2:e190. 10.1371/journal.pgen.0020190 17194218 10.1371/journal.pgen.0020190 PMC1713260

[CR88] Patterson N, Moorjani P, Luo Y et al (2012) Ancient admixture in human history. Genetics 192(3):1065–1093. 10.1534/genetics.112.145037 22960212 10.1534/genetics.112.145037 PMC3522152

[CR89] Pereira V, Gusmão L, Valente C et al (2012) Refining the genetic portrait of Portuguese Roma through X-chromosomal markers. Am J Phys Anthropol 148:389–394. 10.1002/ajpa.22061 22576185 10.1002/ajpa.22061

[CR90] Pérez J, Hochroth L (2007) History of a tragedy: the expulsion of the Jews from Spain. University of Illinois Press

[CR91] Picard toolkit (2019) Picard toolkit. Broad Institute, GitHub repository

[CR92] Pickrell JK, Patterson N, Loh P-R et al (2014) Ancient West Eurasian ancestry in Southern and Eastern Africa. Proc Natl Acad Sci 111:2632–2637. 10.1073/pnas.1313787111 24550290 10.1073/pnas.1313787111 PMC3932865

[CR93] Pohoryles Y (2018) The Jewish-Romani connection: Are Gypsies descendants of tribe of Simeon? Ynetnews. Retrieved from https://www.ynetnews.com/articles/0,7340,L-5248902,00.html

[CR94] Price AL, Patterson NJ, Plenge RM et al (2006) Principal components analysis corrects for stratification in genome-wide association studies. Nat Genet 38:904–909. 10.1038/ng1847 16862161 10.1038/ng1847

[CR95] Pym R (2007) The Gypsies of early modern Spain. Springer

[CR96] Rai N, Chaubey G, Tamang R et al (2012) The phylogeography of Y-Chromosome haplogroup H1a1a-M82 reveals the likely Indian origin of the European Romani populations. PLoS ONE 7:e48477. 10.1371/journal.pone.0048477 23209554 10.1371/journal.pone.0048477 PMC3509117

[CR97] Reich D, Thangaraj K, Patterson N et al (2009) Reconstructing Indian population history. Nature 461:489–494. 10.1038/nature08365 19779445 10.1038/nature08365 PMC2842210

[CR98] Sánchez DM (2022) Historia Del Pueblo Gitano En España. Los Libros De La Catarata

[CR99] Severson AL, Carmi S, Rosenberg NA (2019) The effect of consanguinity on Between-Individual Identity-by-Descent sharing. Genetics 212:305–316. 10.1534/genetics.119.302136 30926583 10.1534/genetics.119.302136 PMC6499533

[CR100] Silvestre J (2005) Internal migrations in Spain, 1877–1930. Eur Rev Econ Hist 9:233–265. 10.1017/S1361491605001462

[CR101] Soyer F (2007) The Persecution of the Jews and Muslims of Portugal: King Manuel I and the End of Religious Tolerance (1496-7). BRILL

[CR102] Tarnovschi D, Preoteasa AM, Pamporov A et al (2012) Roma from Romania, Bulgaria, Italy and Spain between social inclusion and migration. Soros Foundation Romania, Bucharest

[CR103] Tartakoff P (2012) Between Christian and Jew: conversion and inquisition in the crown of Aragon. University of Pennsylvania, pp 1250–1391

[CR104] Universitat Pompeu Fabra (2023) Presentació del projecte El camí del Poble Gitano: una història de diversitat. Available: https://www.youtube.com/watch?v=JRP9So3bnwc

[CR105] Vilà-Valls L, Aizpurua-Iraola J, Casinge S et al (2023) Genomic insights into the population history of the Resande or Swedish travelers. Genome Biol Evol 15:evad006. 10.1093/gbe/evad006 36655389 10.1093/gbe/evad006 PMC9907538

[CR106] Waldman YY, Biddanda A, Dubrovsky M et al (2016) The genetic history of Cochin Jews from India. Hum Genet 135:1127–1143. 10.1007/s00439-016-1698-y 27377974 10.1007/s00439-016-1698-y PMC5020127

[CR107] Wangkumhang P, Greenfield M, Hellenthal G (2022) An efficient method to identify, date, and describe admixture events using haplotype information. Genome Res 32:1553–1564. 10.1101/gr.275994.121 35794007 10.1101/gr.275994.121 PMC9435750

[CR108] Weyrauch WO (2001) Gypsy law: Romani legal traditions and culture. University of California Press

[CR109] Zahra T (2017) Condemned to rootlessness and unable to Budge: Roma, migration panics, and internment in the Habsburg empire. Am Hist Rev 122:702–726

[CR110] Zhou Y, Browning SR, Browning BL (2020) A fast and simple method for detecting Identity-by-Descent segments in Large-Scale data. Am J Hum Genet 106:426–437. 10.1016/j.ajhg.2020.02.010 32169169 10.1016/j.ajhg.2020.02.010 PMC7118582

[CR111] Zou JY, Halperin E, Burchard E, Sankararaman S (2015a) Inferring parental genomic ancestries using pooled semi-Markov processes. Bioinformatics 31:i190–i196. 10.1093/bioinformatics/btv239 26072482 10.1093/bioinformatics/btv239 PMC4765873

[CR112] Zou JY, Park DS, Burchard EG et al (2015b) Genetic and socioeconomic study of mate choice in Latinos reveals novel assortment patterns. Proceedings of the National Academy of Sciences 112:13621–13626. 10.1073/pnas.1501741112 10.1073/pnas.1501741112 PMC4640764 26483472

