# Supplementary material for: The genetic footprint of the European Roma diaspora: evidence from the Balkans to the Iberian Peninsula
Source: Hum Genet. 2025 Mar 17;144(4):463–79. doi: 10.1007/s00439-025-02735-z (PMC12003505; doi:10.1007/s00439-025-02735-z)
Supplement: Supplementary file 1 — Supplementary Material 1 [file 439_2025_2735_MOESM1_ESM.docx]

***The genetic footprint of the European Roma diaspora: Evidence from the Balkans to the Iberian Peninsula***

*Giacomo F. Ena1 (ORCID: 0000-0002-7749-5182), Aaron Giménez2, Annabel Carballo-Mesa3, Petra Lišková4,5 (ORCID: 0000-0003-3519-643X), Marcos Araújo Castro e Silva1 (ORCID: 0000-0002-9873-3717), David Comas1 (ORCID: 0000-0002-5075-0956), **

*1 Institut de Biologia Evolutiva (CSIC-UPF), Universitat Pompeu Fabra, Departament de Medicina i Ciències de la Vida, Barcelona*

*2 Facultat de Sociologia, Universitat Autònoma de Barcelona, Barcelona, Spain*

*3 Facultat de Geografia i Història, Universitat de Barcelona, Barcelona, Spain*

*4 Department of Paediatrics and Inherited Metabolic Disorders, First Faculty of Medicine, Charles University and General University Hospital in Prague, Prague, Czech Republic*

*5 Department of Ophthalmology, First Faculty of Medicine, Charles University and General University Hospital in Prague, Prague, Czech Republic*

** Corresponding author*

*E-mail:* [*david.comas@upf.edu*](mailto:david.comas@upf.edu)

***Supplementary Figures***


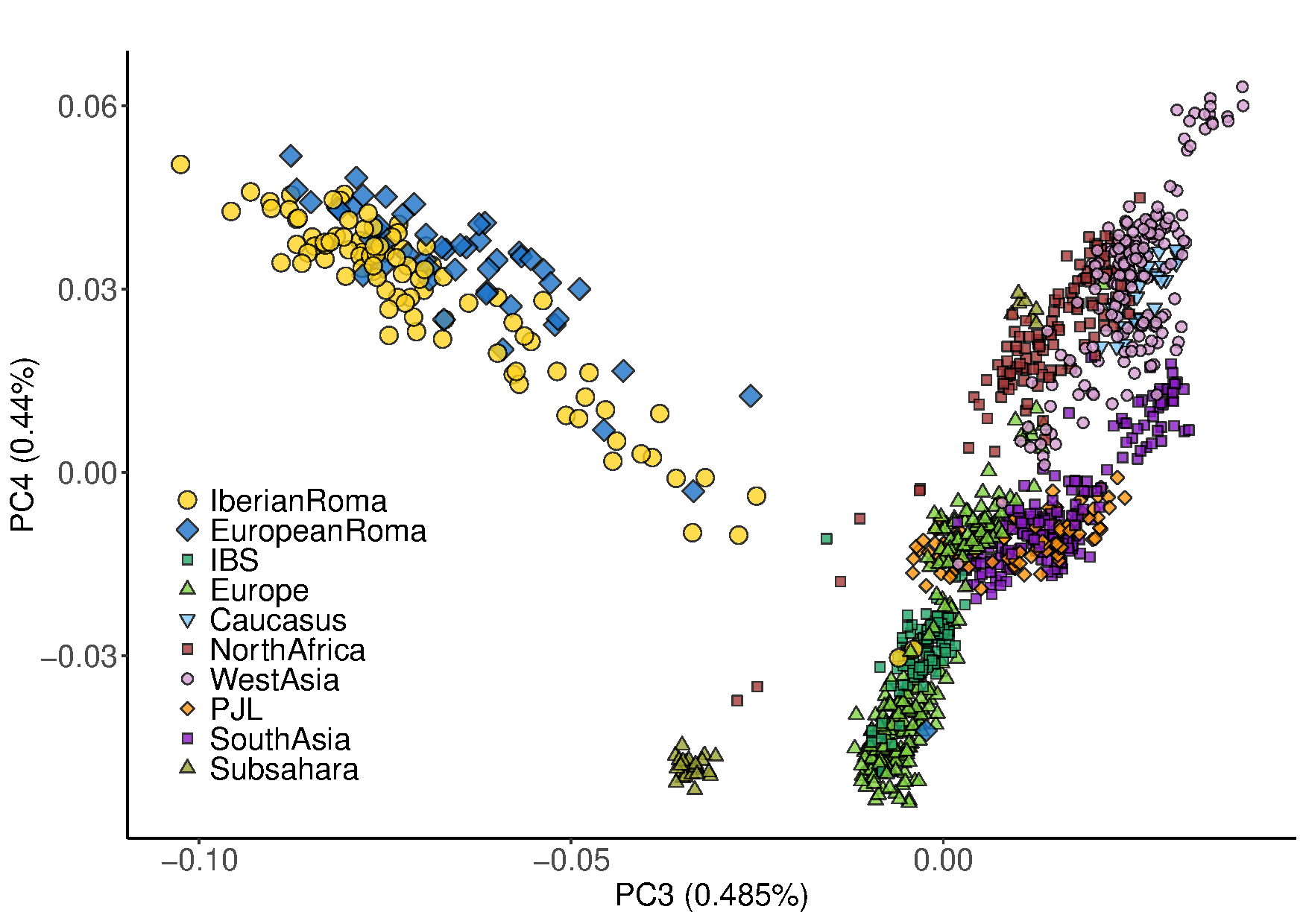


***Supplementary Figure 1*** *Principal Component Analysis on an Afro-Eurasian scale.*

*PCA displaying Principal Components 3 and 4, including all Roma samples and references analysed in this study.*

***
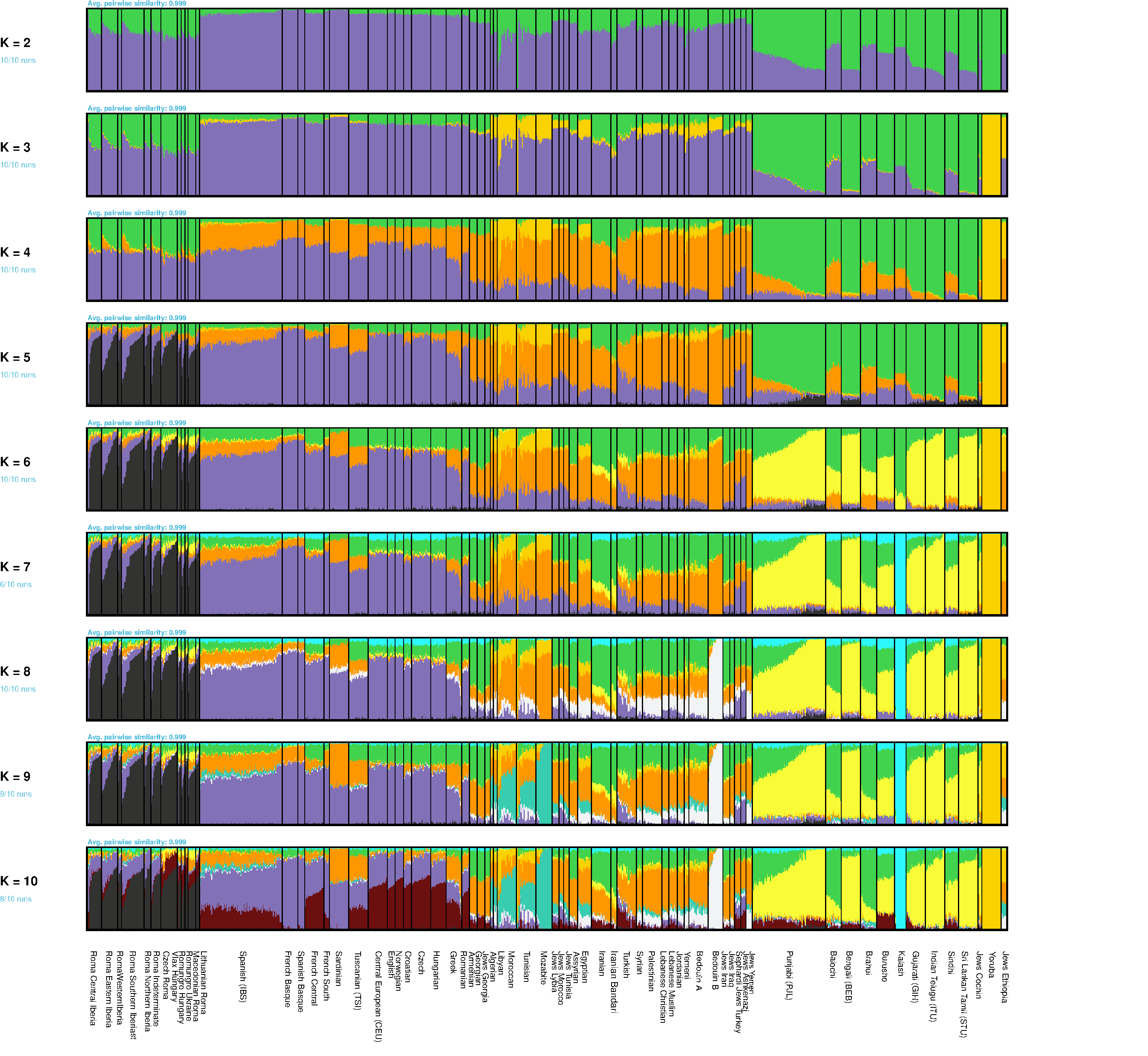
***

**Supplementary Figure 2 ADMIXTURE results on the whole dataset.**

*Results from all ADMIXTURE runs performed on the main dataset, with values of K ranging from 2 to 10.*

*
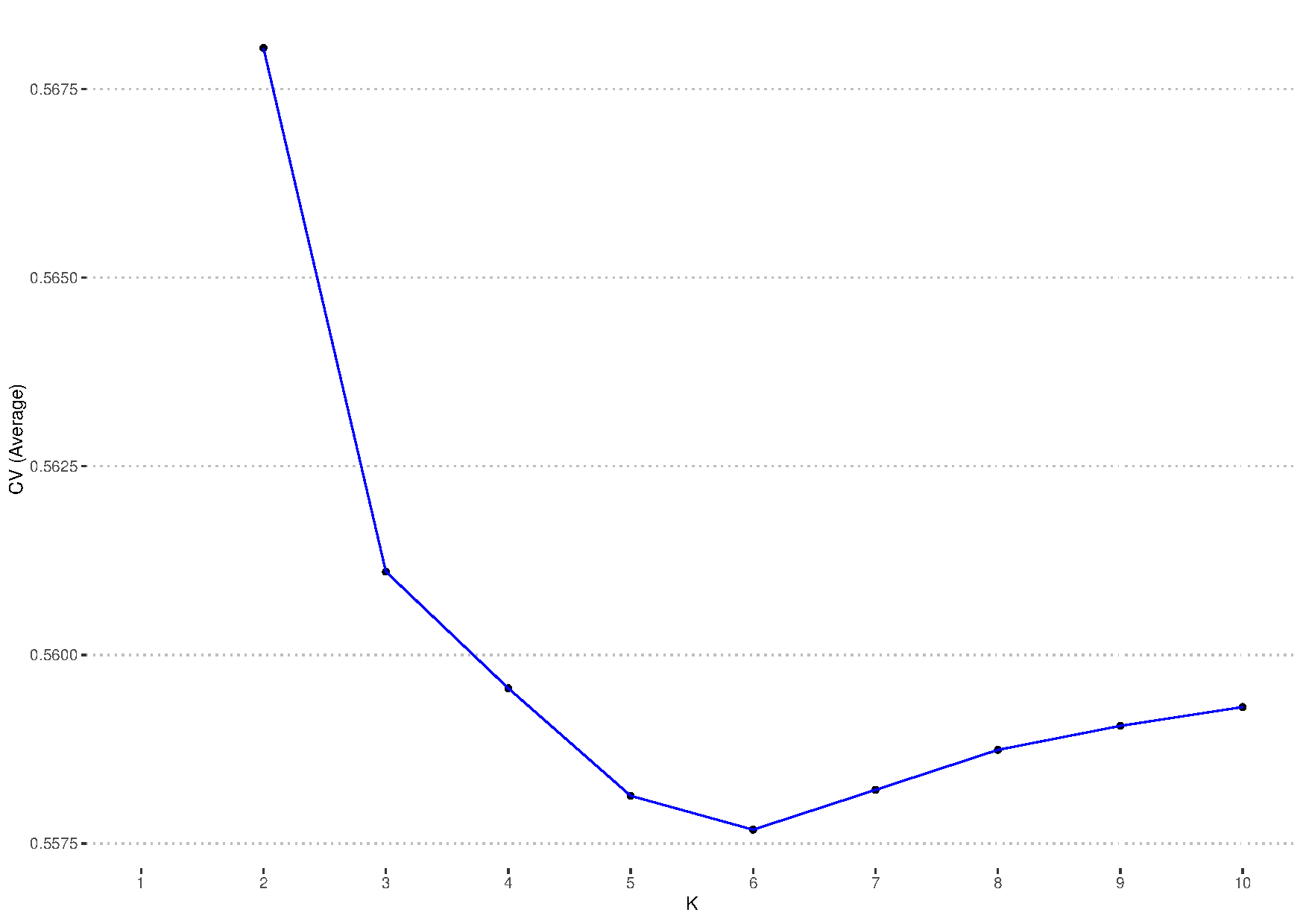
*

**Supplementary Figure 3 Plot of cross-validation values for ADMIXTURE on the whole dataset.**


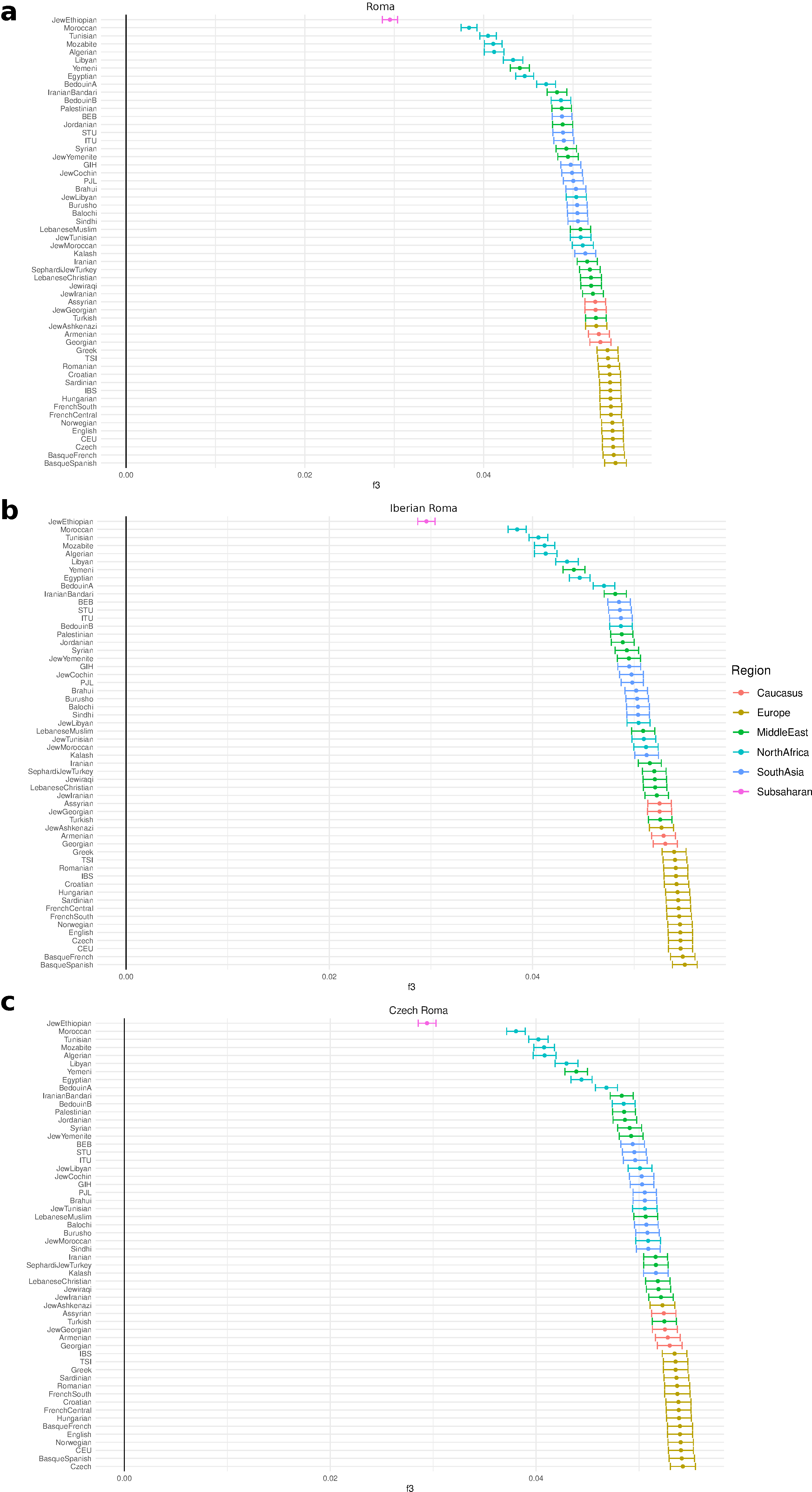


**Supplementary Figure 4 *f****3* outgroup statistical test for admixture.

*(a) f3 outgroup test in the form f3(Yoruba, Roma, Reference), incorporating all reference populations. (b) f3 outgroup test in the form f3(Yoruba, Iberian Roma, Reference), incorporating all reference populations. (c) f3 outgroup test in the form f3(Yoruba, Czech Roma, Reference), incorporating all reference populations.*

*
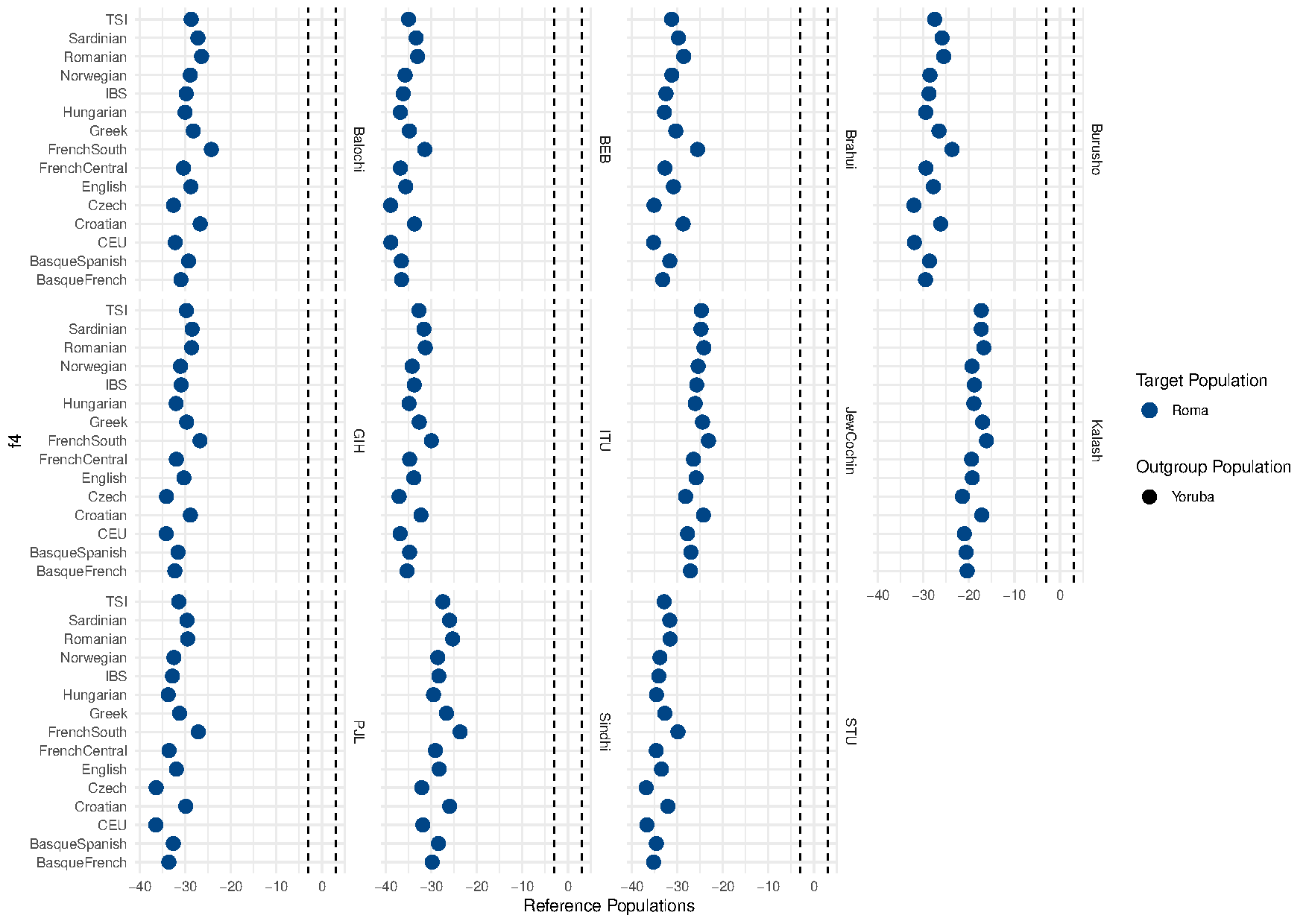
*

**Supplementary Figure 5 *f****4* statistical test for shared drift.

*f4 test in the form f4(Yoruba, Roma, reference, reference), including only references from Europe and South Asia.*

**
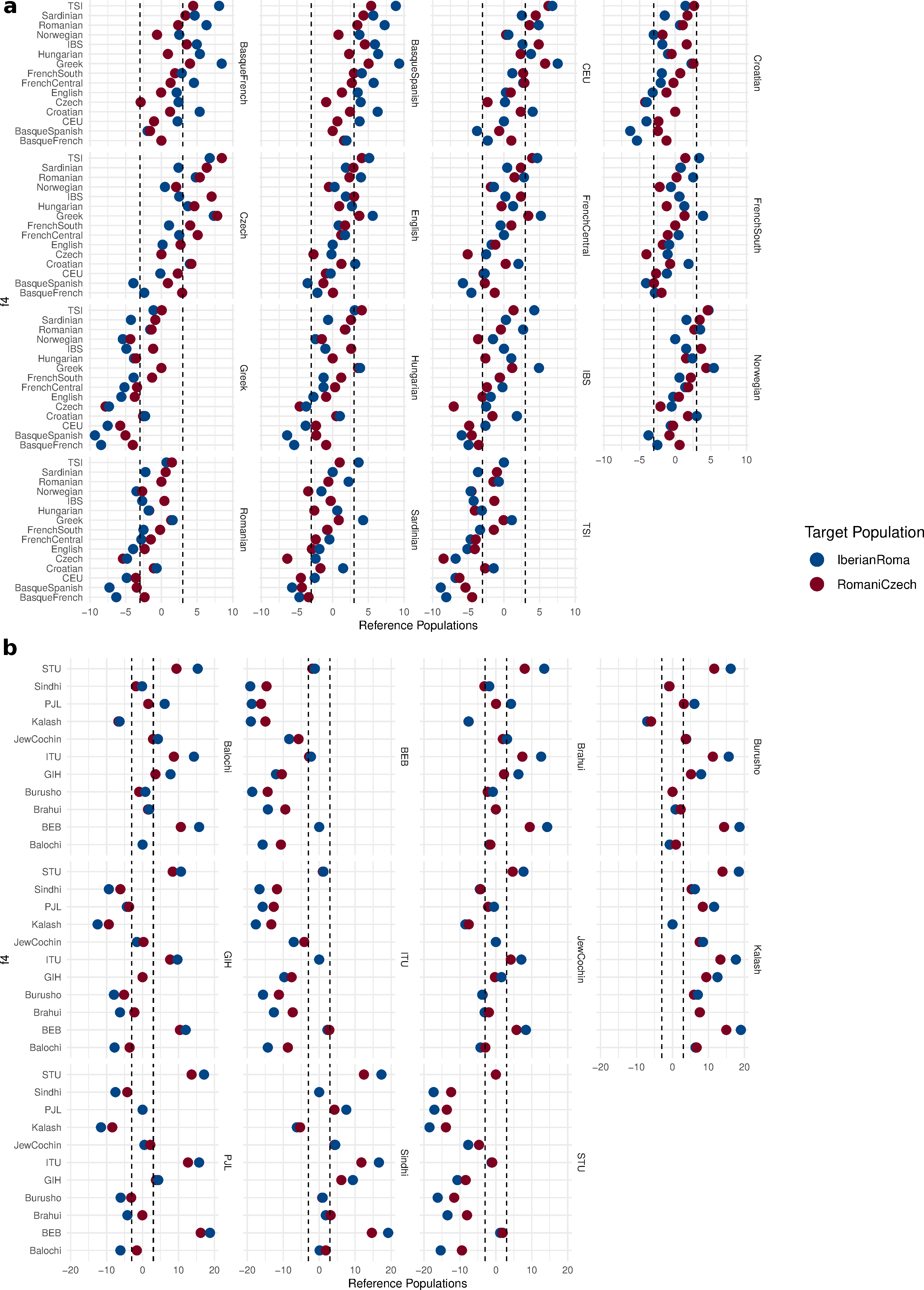
**

**Supplementary Figure 6 *f****4* statistical test for shared drift.

*(a) f4 test including only the European reference populations. (b) f4 test including only the Asian reference populations.*

**
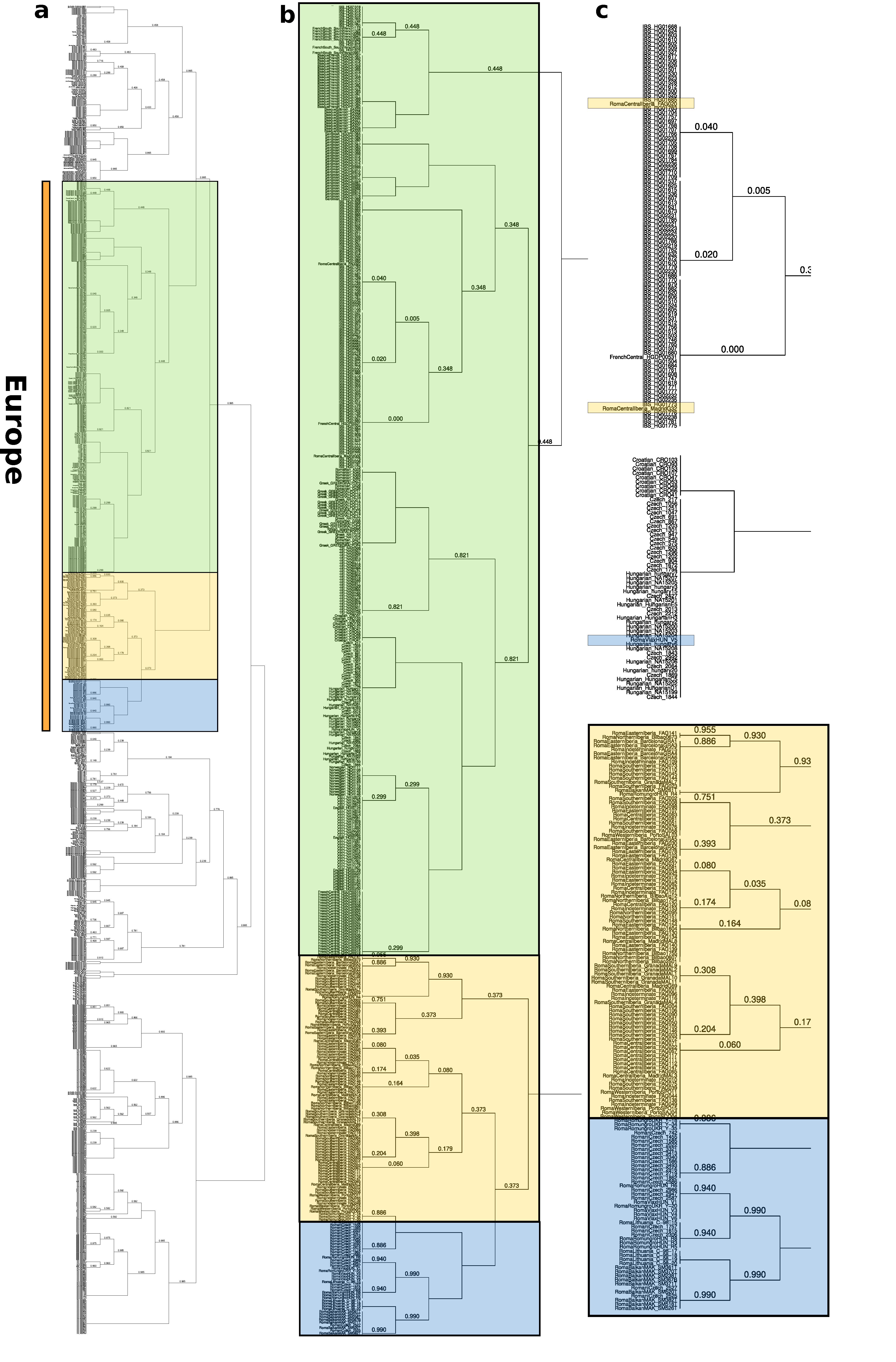
**

**Supplementary Figure 7** Composition of the fineSTRUCTURE dendrogram.

*(a) Whole-tree: European populations are shown in green, the Iberian Roma branch in yellow, and the other European Roma branch in blue. (b) Detailed view of the Europe macro-branch, highlighting the three Roma individuals that cluster outside the Roma macro-branches. (c) Details of the Roma macro-branches, including the section containing the three separated Roma individuals.*


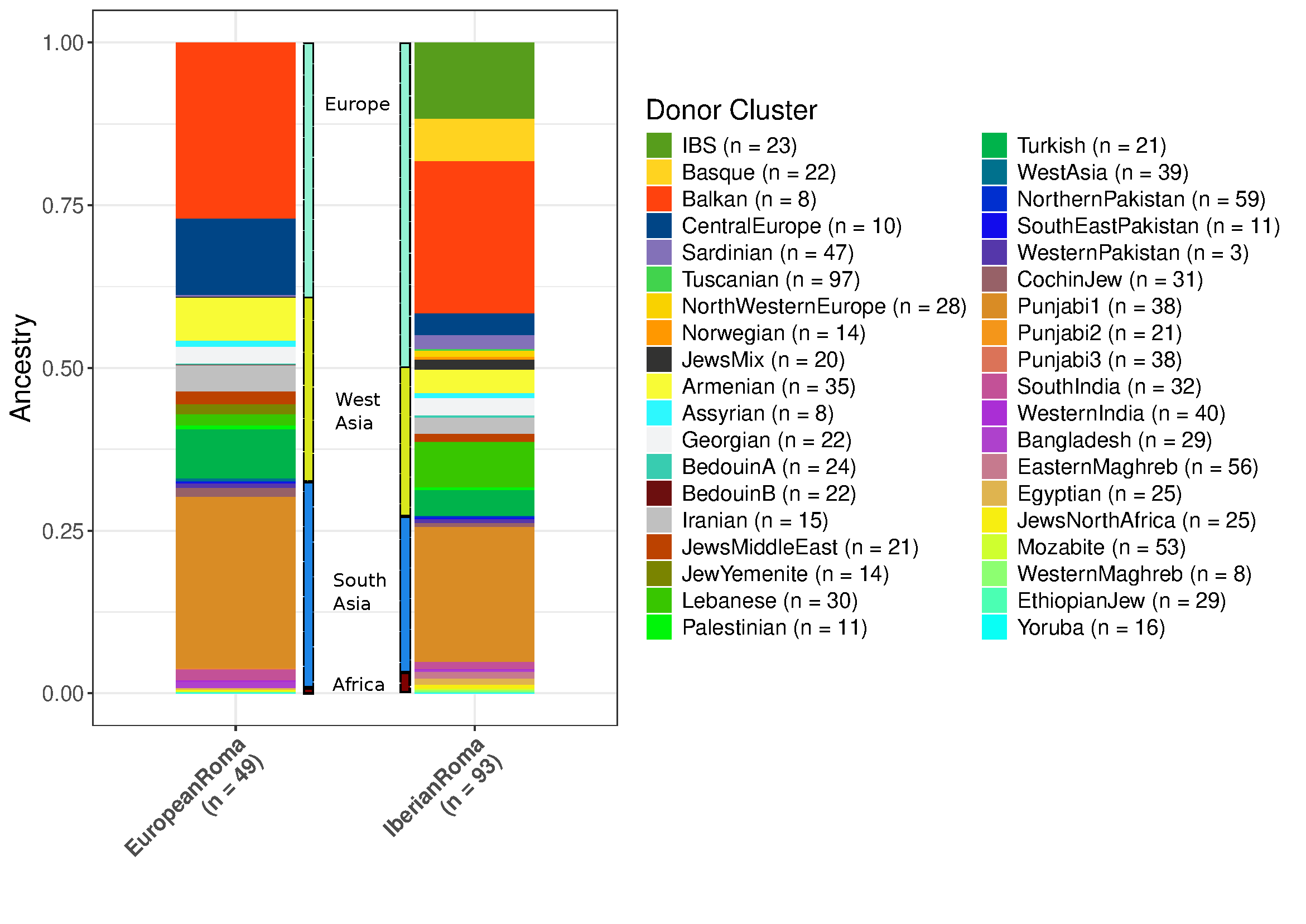


**Supplementary Figure 8** Composition of the Roma genetic profile based on NNLS analysis. *NNLS results derived from the classification of Roma populations based on genetic clustering. The analysis grouped all Roma individuals into two distinct clusters.*

*
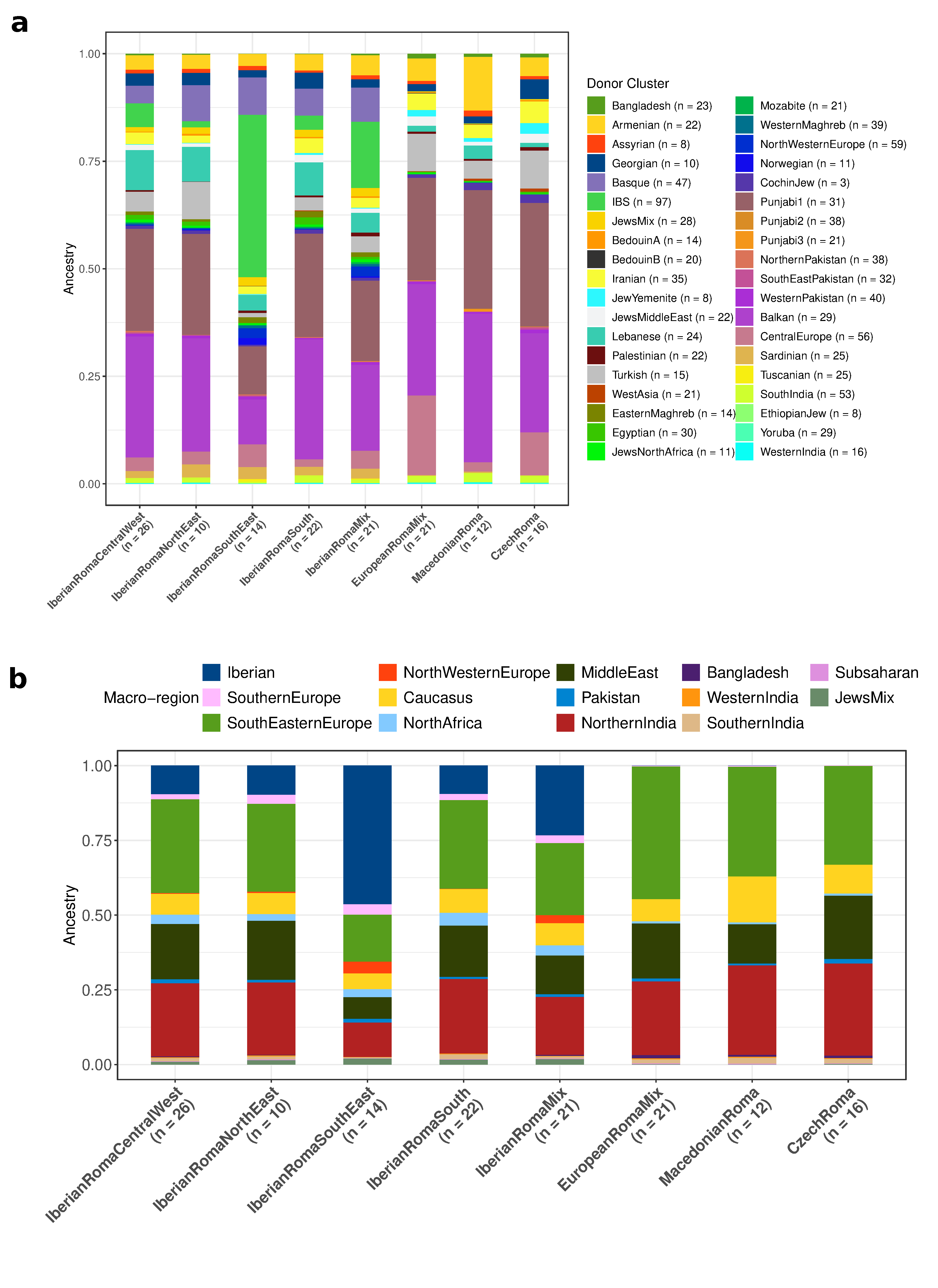
*

**Supplementary Figure 9 R**esults of the NNLS analysis on the subclusters.

*(a) NNLS results categorised by genetic cluster, grouped by donor cluster. (b) NNLS results categorised by genetic cluster, grouped by donor macro-region.*

**
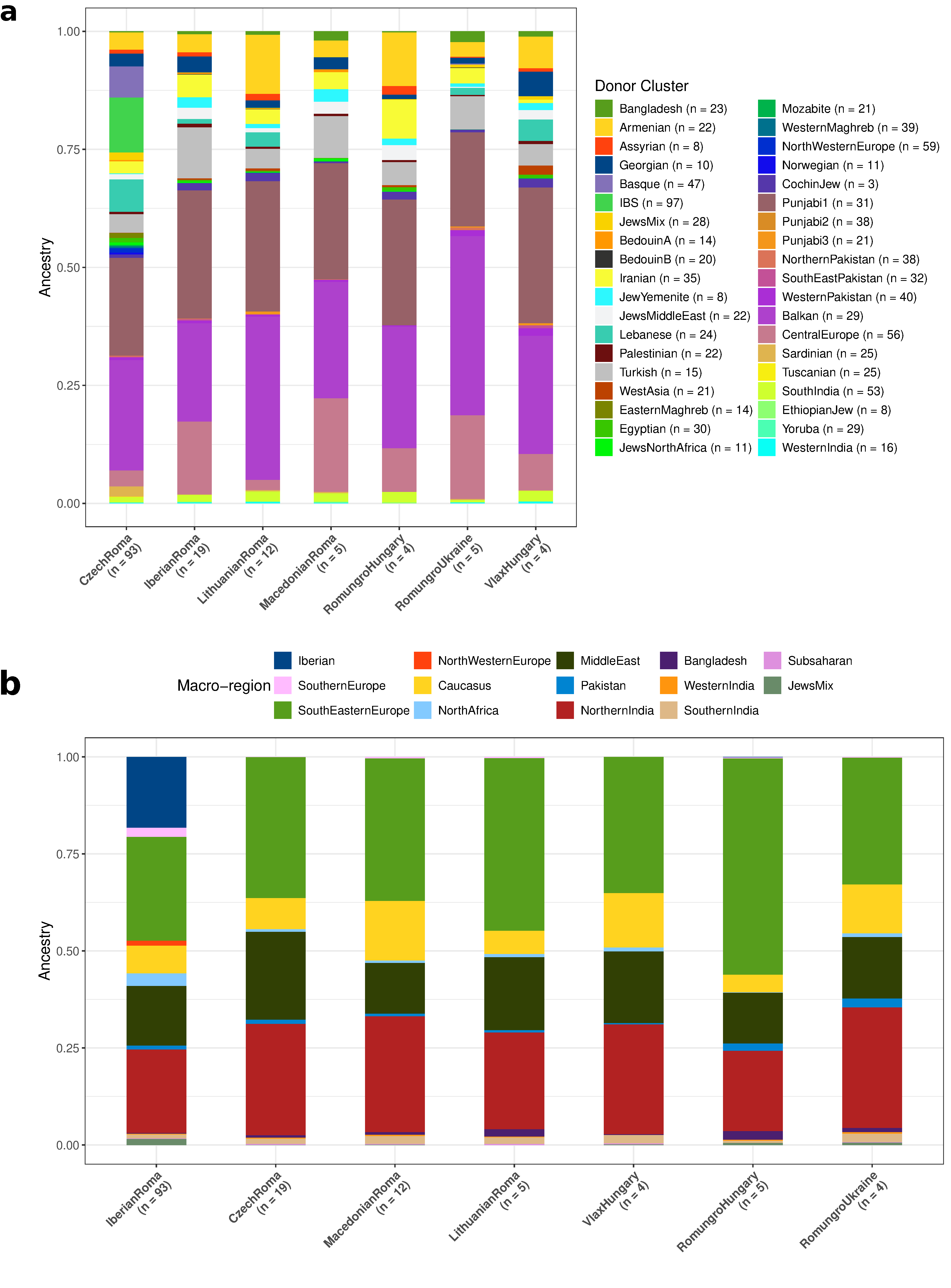
**

**Supplementary Figure 10** Results of the NNLS analysis on geographical regions.

*(a) NNLS results grouped by donor clusters. (b) NNLS results grouped by donor macro-region.*

**S**
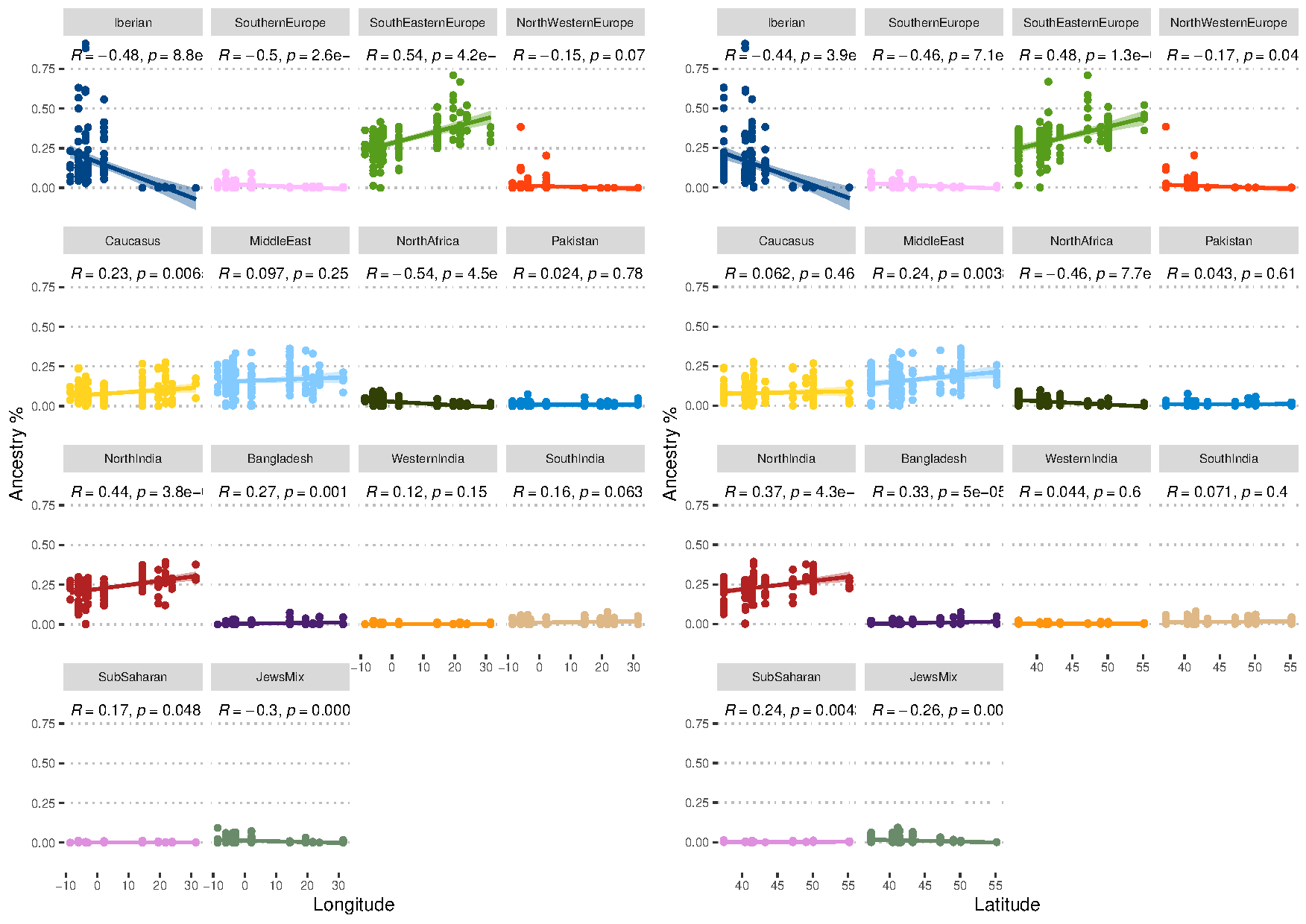
**upplementary Figure 11** Linear regression plot and Pearson correlation coefficients related to the gradient in NNLS ancestry components.

*Pearson correlation analysis based on longitude and latitude, conducted across all donor clusters grouped into macro-regions for each Roma individual.*


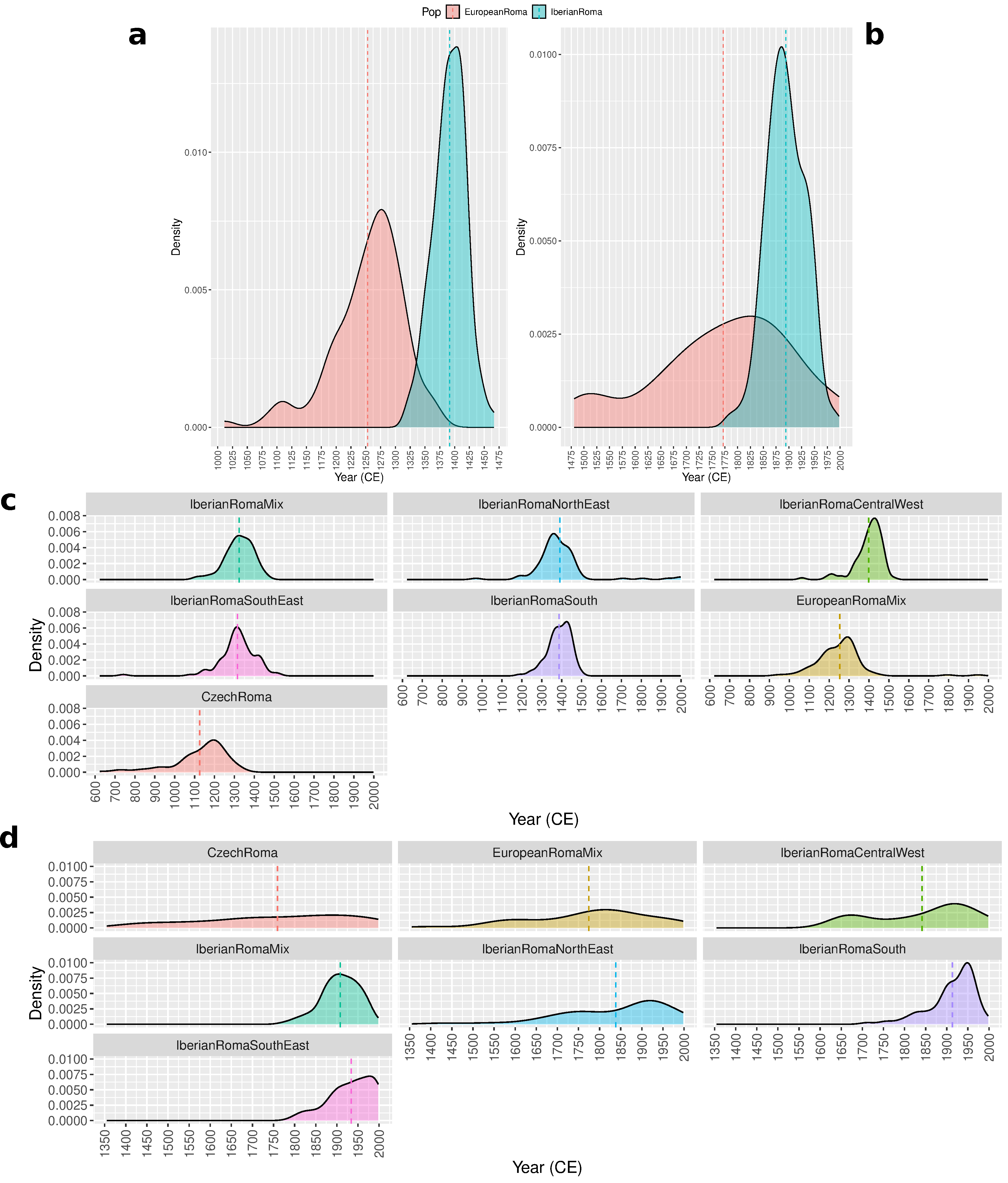
**Supplementary Figure 12** Bootstrap analysis of the fastGLOBETROTTER estimated dates for recipient genetic clusters. **Vertical lines represent the average admixture time for each cluster, with dates expressed in years (CE).**

*(a) Bootstrap analysis of Date 1 for the main Roma clusters. (b) Bootstrap analysis of Date 2 for the main Roma clusters. (c) Bootstrap analysis of Date 1 for subclusters within the main Roma clusters. (d) Bootstrap analysis of Date 2 for subclusters within the main Roma clusters.*

**
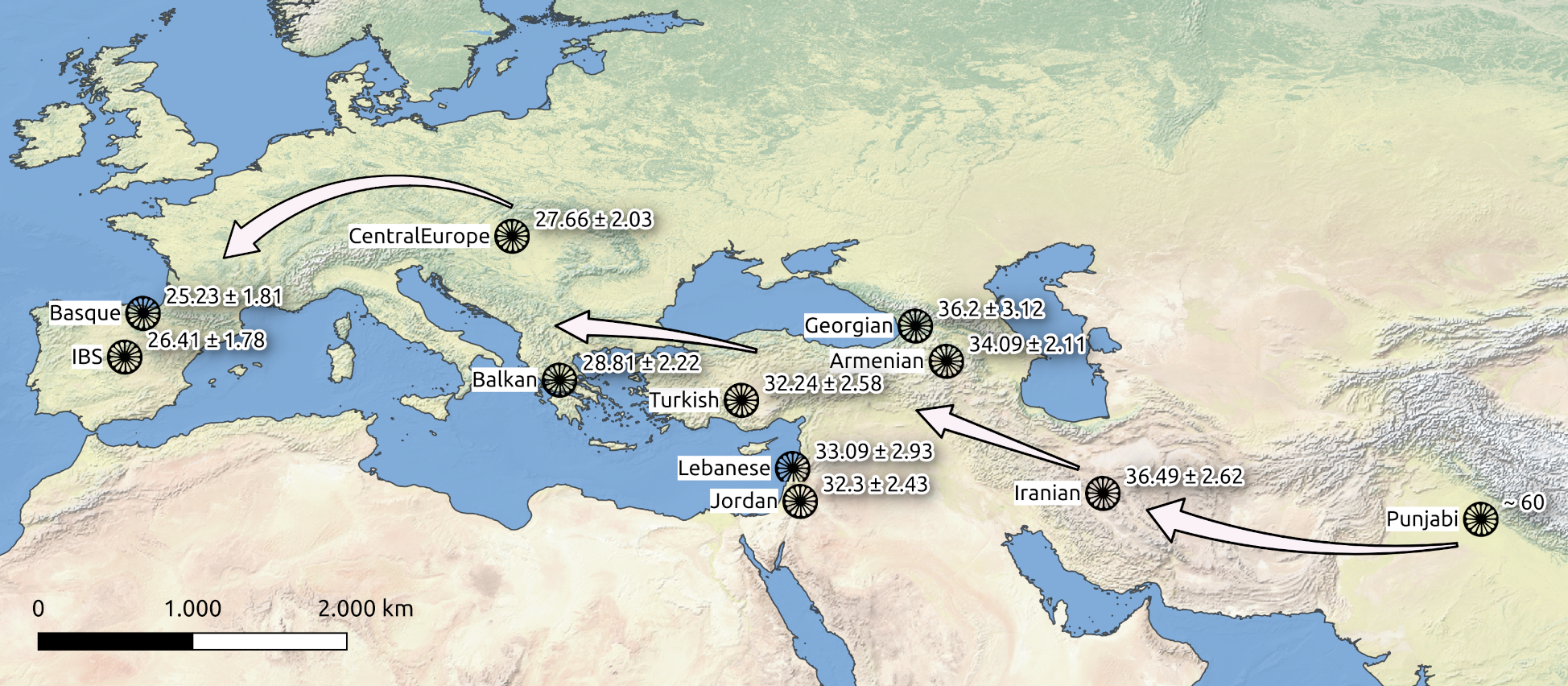
**

**Supplementary Figure 13** Migration of the Roma diaspora.

*Map illustrating the inferred admixture events identified by MALDER and proposed migration routes across the Roma diaspora. The starting point is based on historical estimations.*


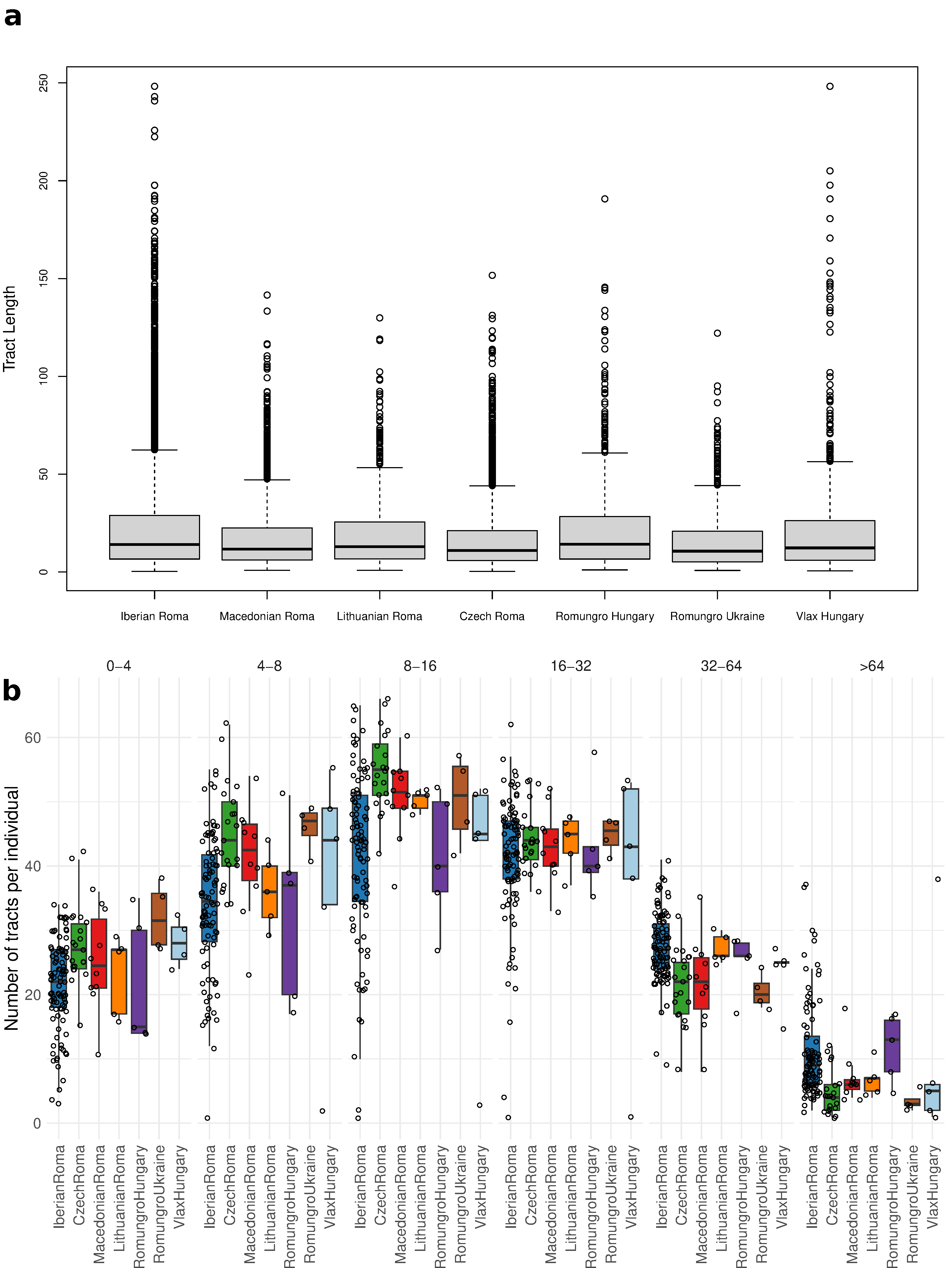
**Supplementary Figure 14** Distribution of European local ancestry tracts.

*(a) Distribution of local ancestry tracts across each Roma population. (b) Distribution of local ancestry tract numbers by length category for each Roma population.*

**
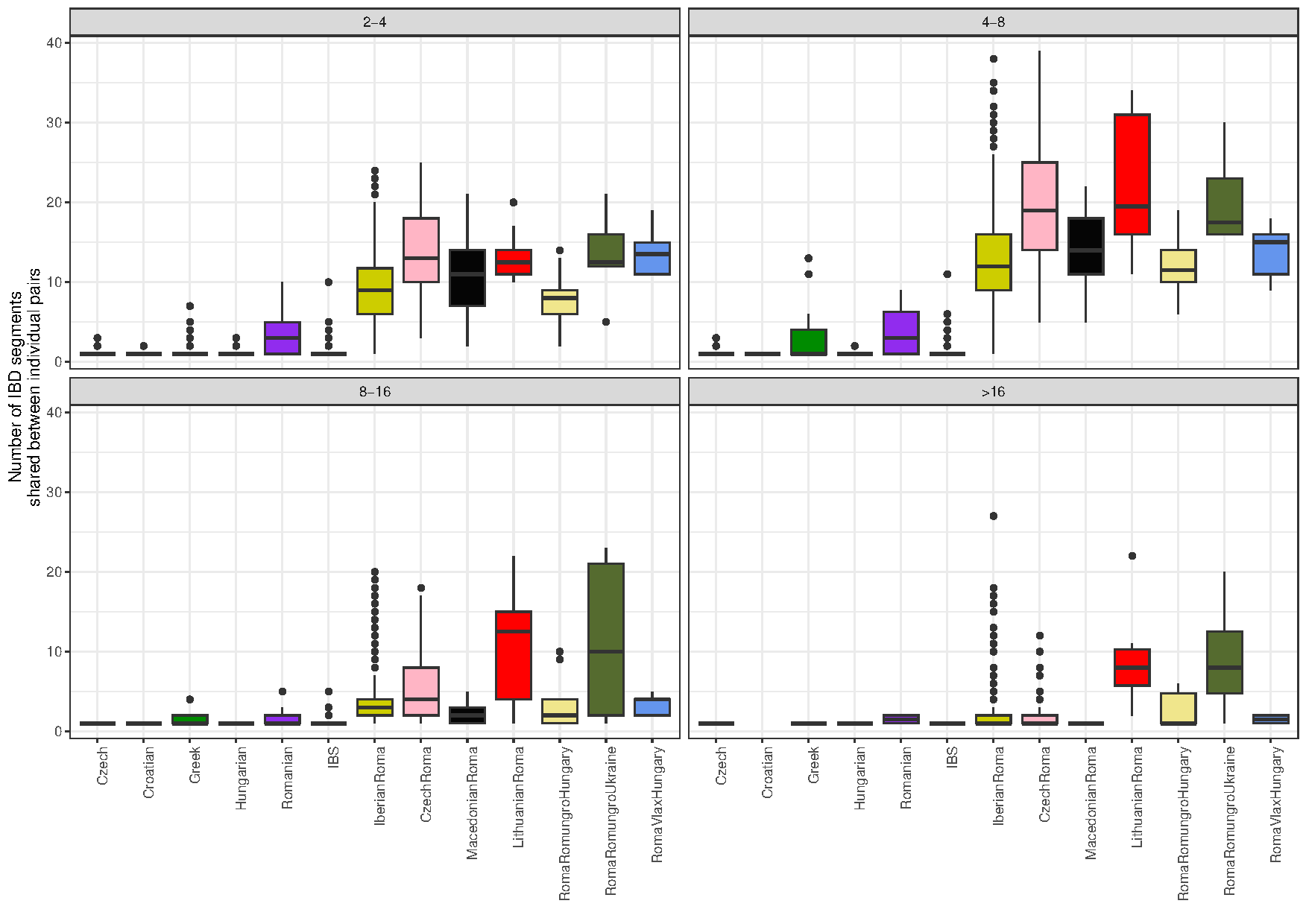
**

**Supplementary Figure 15 Distribution of shared IBD segments within populations by length category in Roma and non-Roma reference populations.**

**
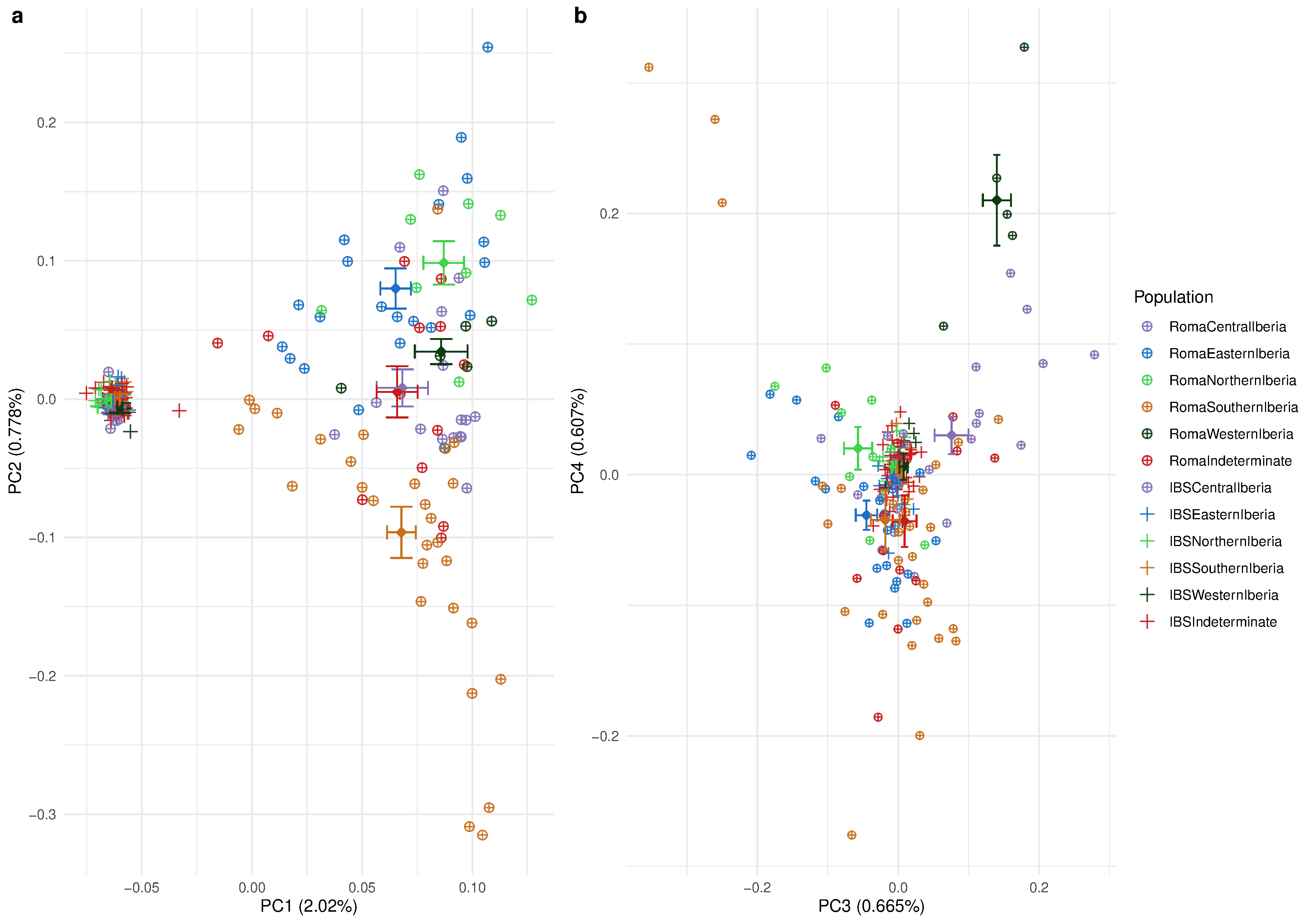
**

**Supplementary Figure 16** Principal Component Analysis on the Iberian dataset.

*PCA conducted using the Iberian dataset. We calculated the confidence intervals at a significance level of 95% for each centroid, representing the average of each principal component.*

*
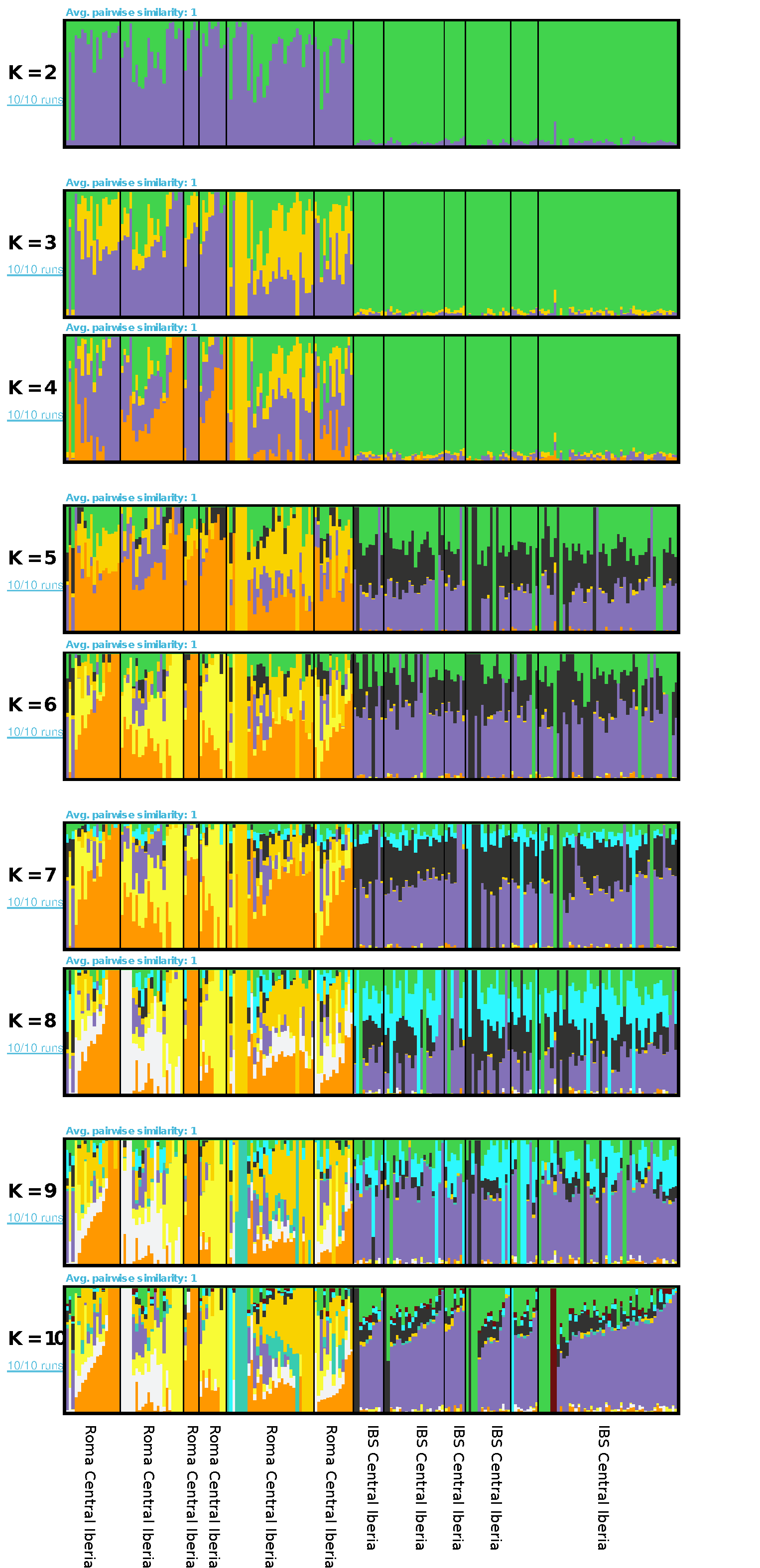
*

**Supplementary Figure 17** ADMIXTURE results on the Iberian dataset.

*Results from ADMIXTURE analyses using the Iberian dataset for all performed runs (K = 2 to K = 10).*

*
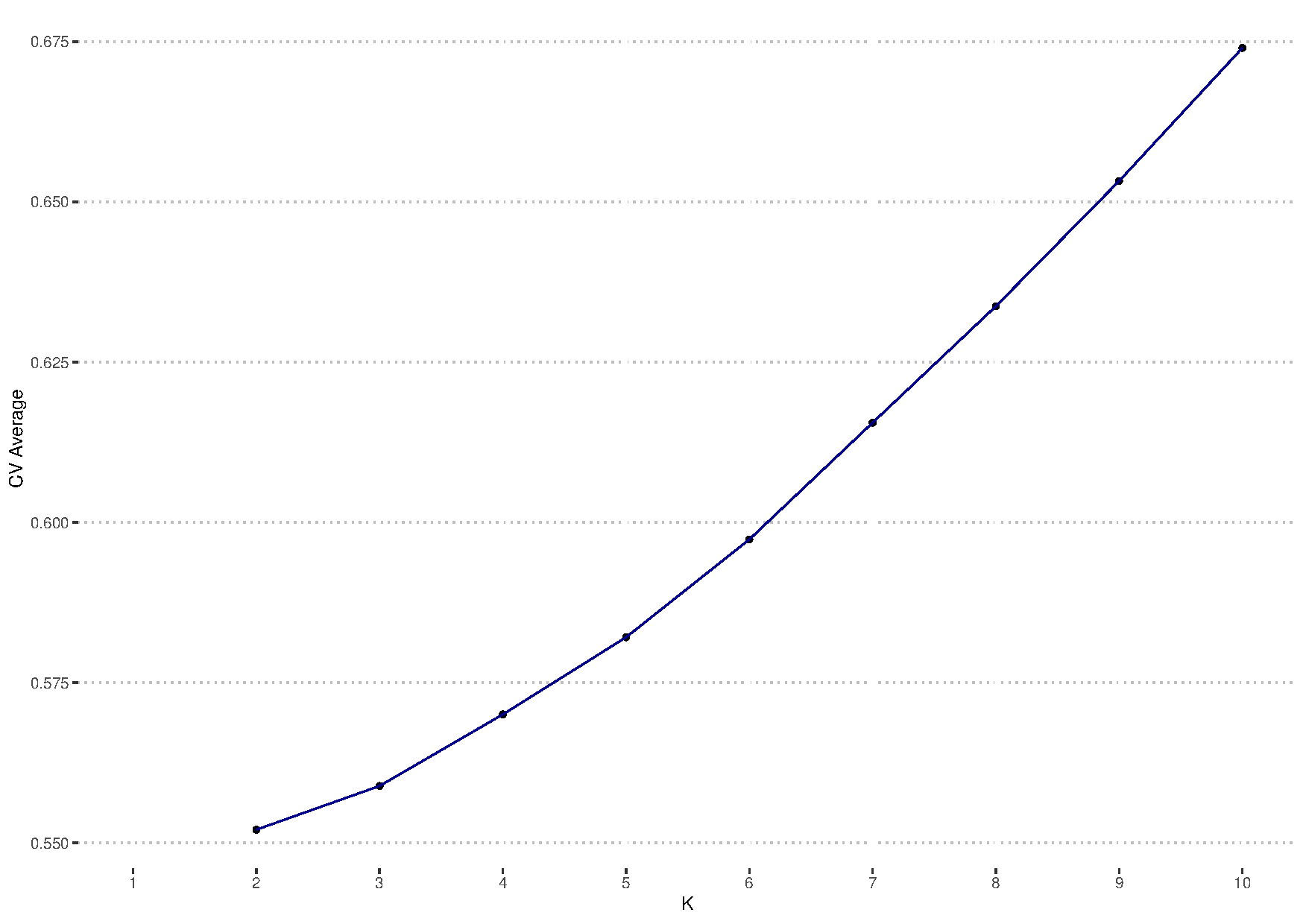
*

**Supplementary Figure 18** Plot of cross-validation values for the ADMIXTURE on the Iberian dataset.

*
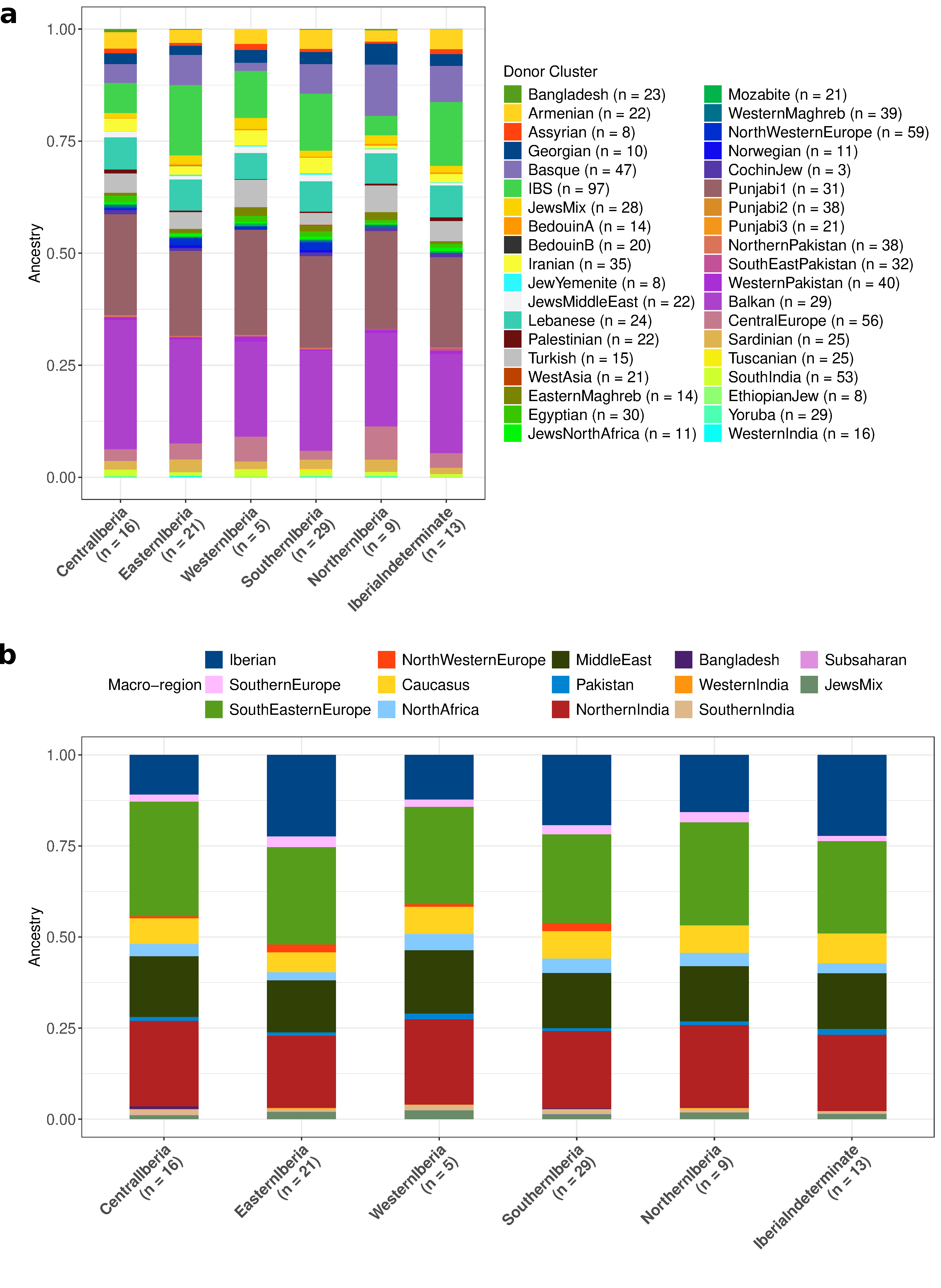
*

*S***upplementary Figure 19 R**esults of the NNLS analysis on the Iberian dataset.

*(a) NNLS performed by genetic cluster, with results grouped by donor macro-region. (b) NNLS performed by genetic cluster, with results grouped by donor cluster.*

**
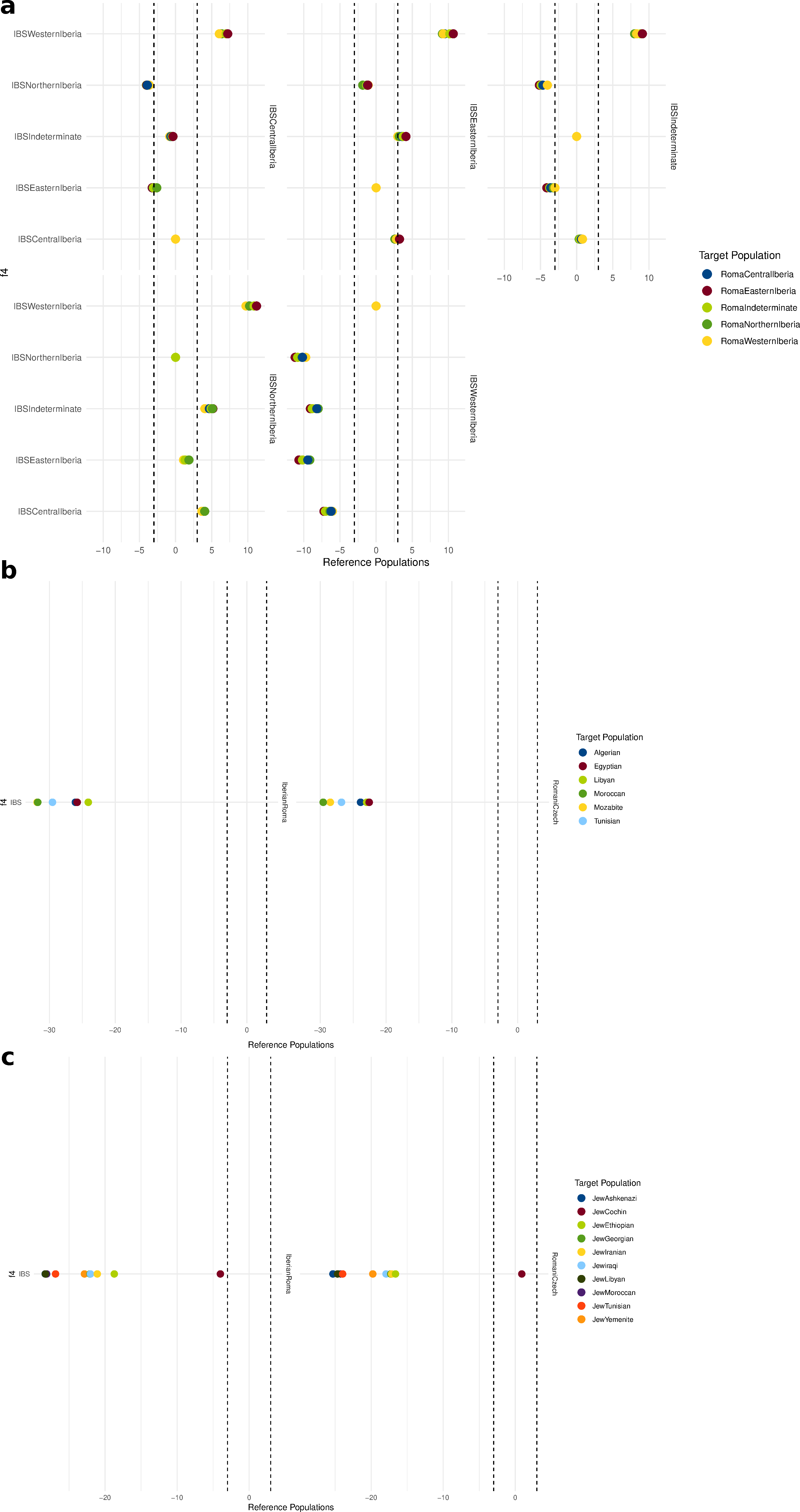
**

**Supplementary Figure 20 *f4*** statistical tests for admixture.

*(a) f4 test on the Iberian dataset. (b)* *f4 test for admixture with North African populations. (c) f4 test for admixture with Jewish populations.*

*
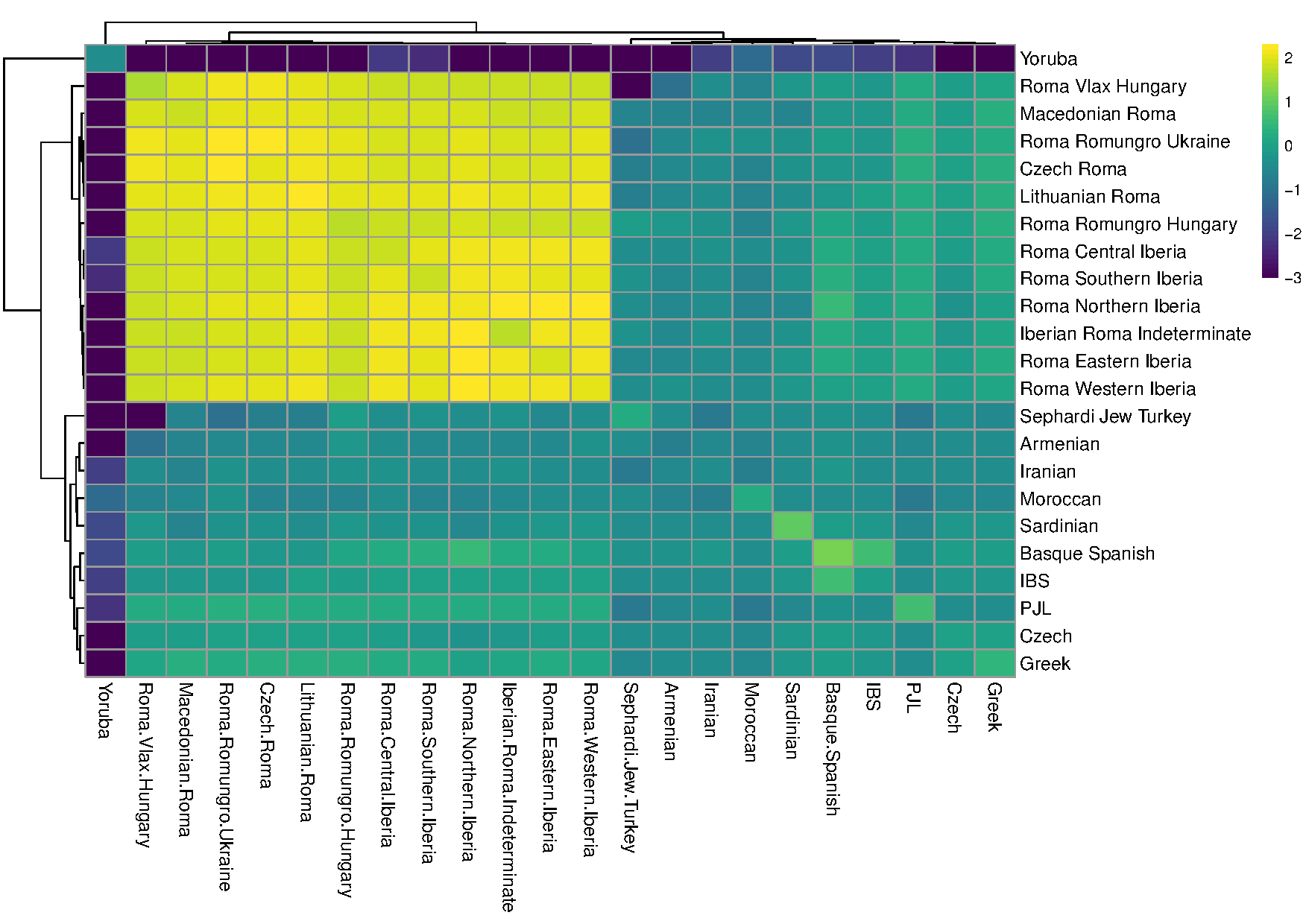
*

**Supplementary Figure 21** Heatmap of pairwise shared IBD segments in the Roma population and reference populations.

*The heatmap displays IBD segment sharing between pairs of populations included in the present study. Reference populations were selected to represent each macro-region. Data is presented on a logarithmic scale.*

*
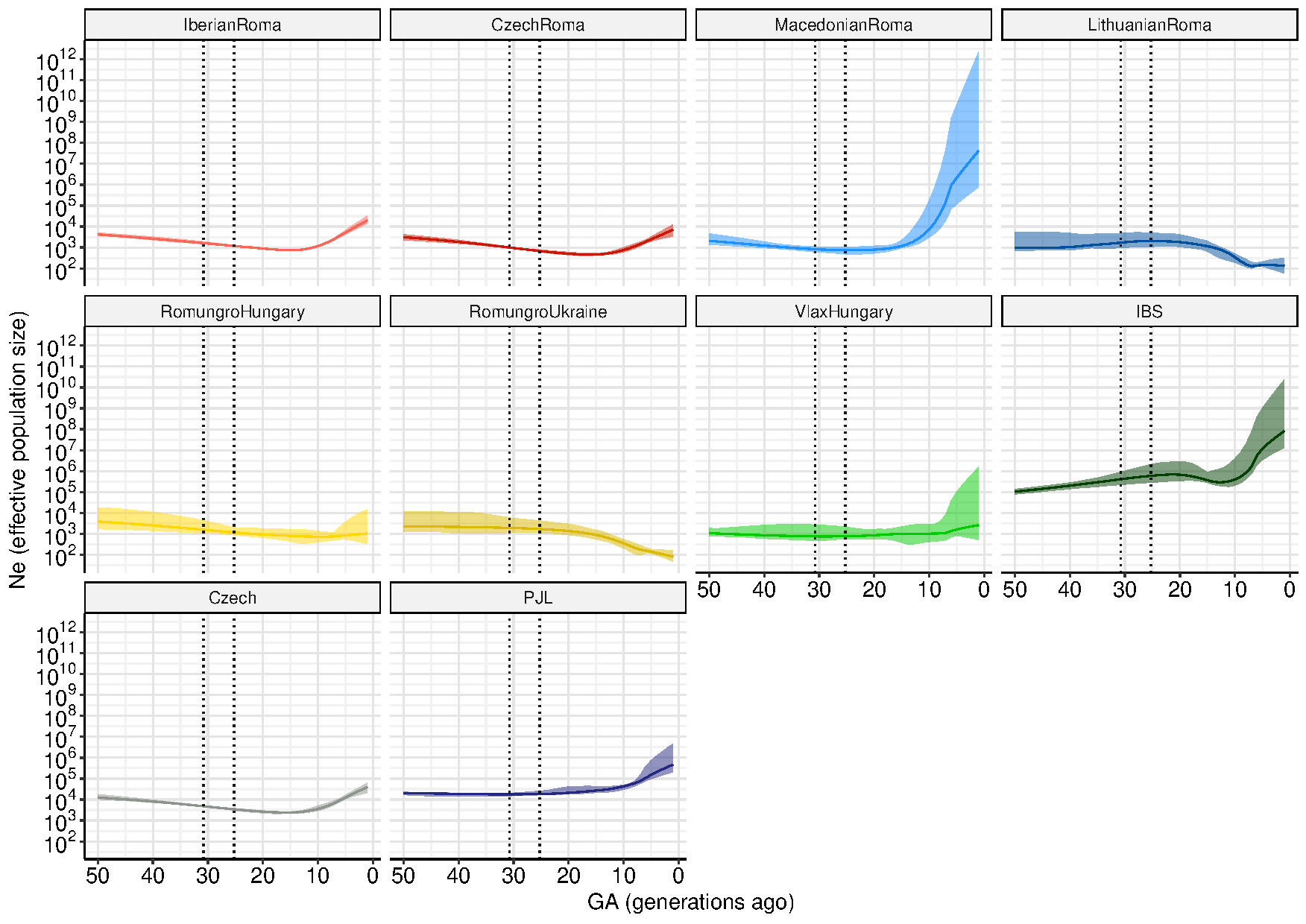
*

**Supplementary Figure 22** Effective population size trend for each Roma population over the past 50 generations.

*The vertical lines indicate the dates of the Roma's arrival in Europe (circa 31 generations ago) and in Spain (circa 25 generations ago), as inferred by fastGLOBETROTTER analysis. The horizontal lines represent the detected effective population size (Ne) values for each generation, with shaded areas indicating the confidence intervals for these values.*

**
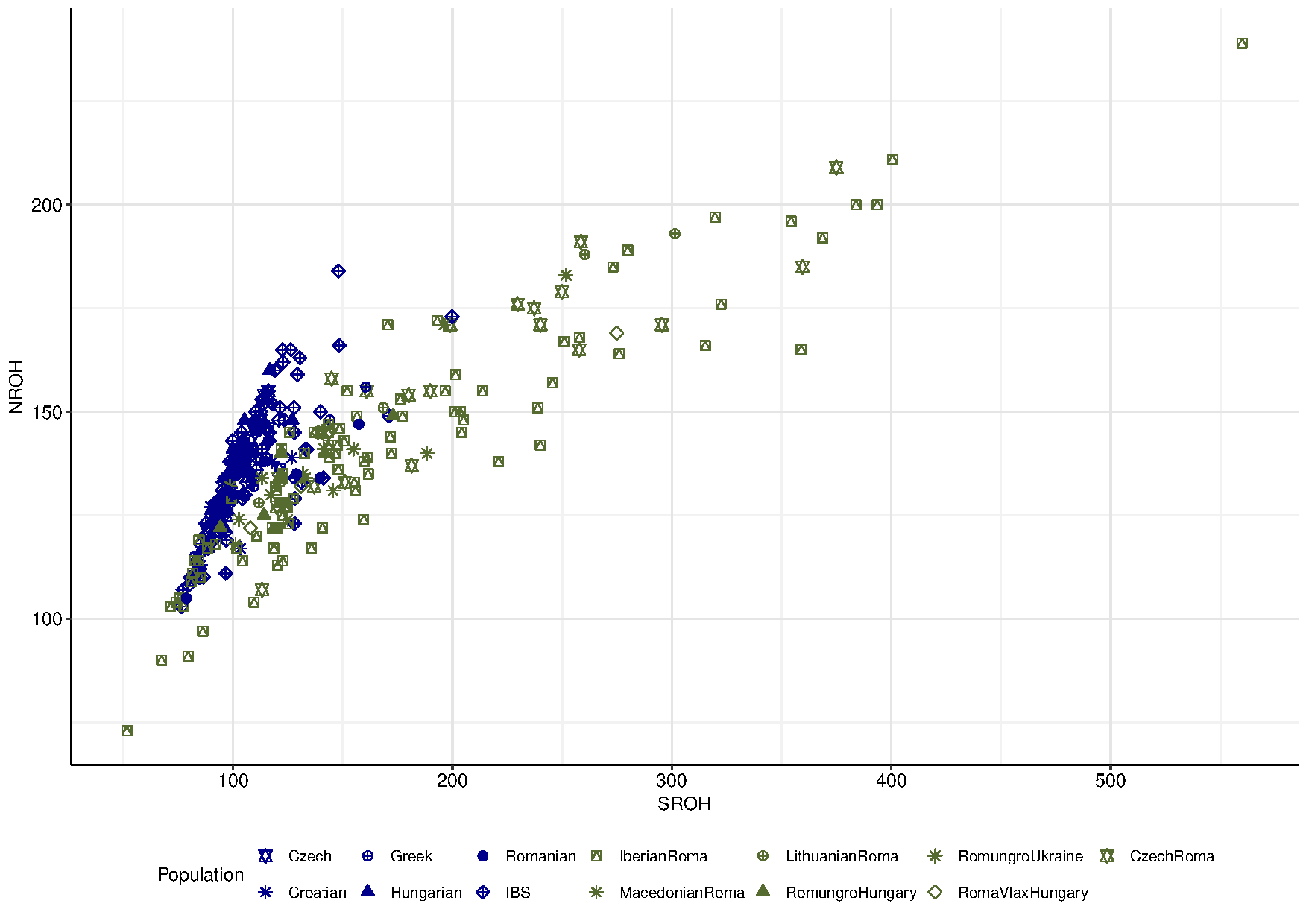
**

**Supplementary Figure 23 Cumulative number (NROH) and size (SROH) of ROHs by individual for Roma and a limited set of references.**

**
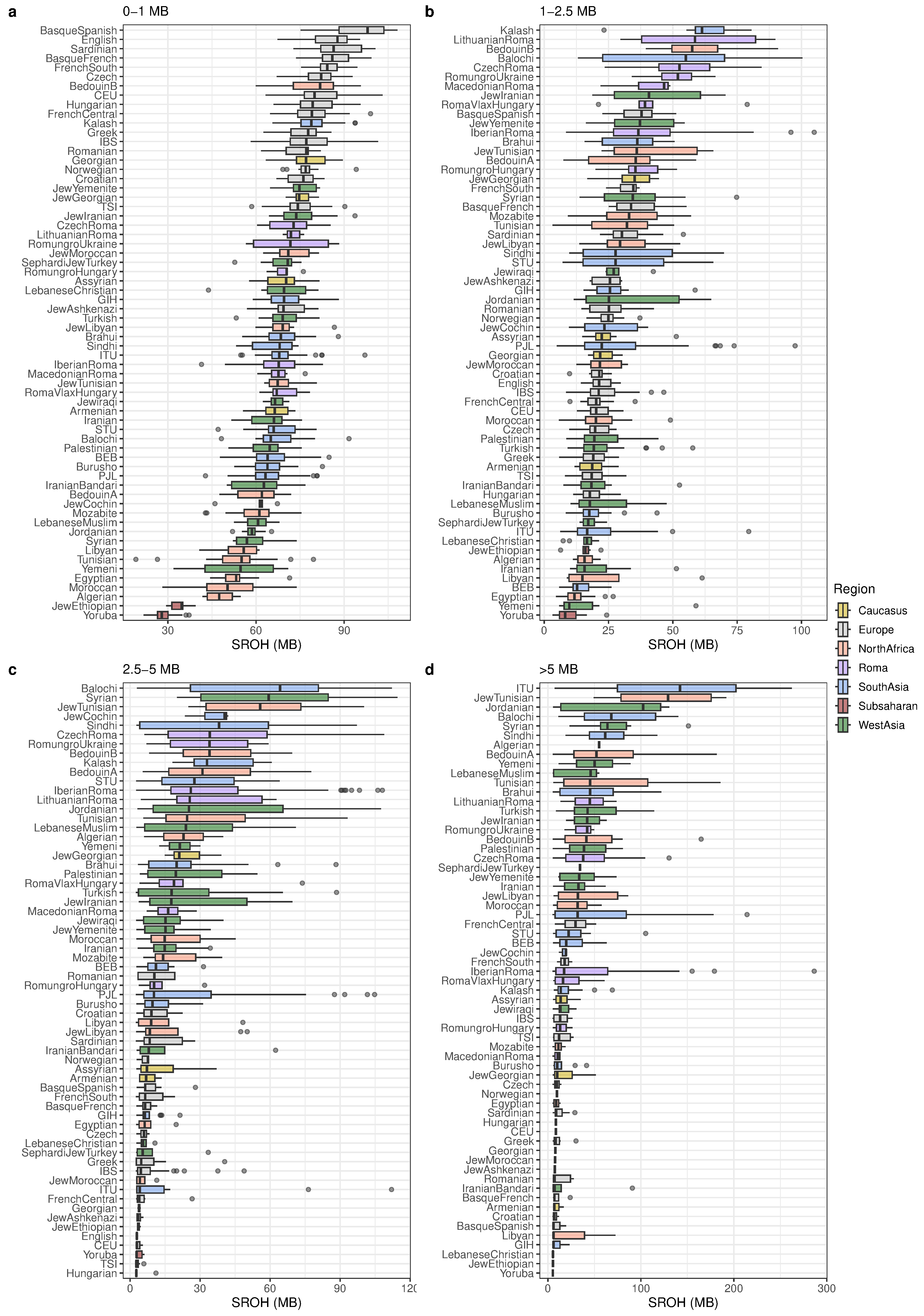
**

**Supplementary Figure 24 (a-d) Average number of ROHs per individual by length class in each population of the dataset.**

**
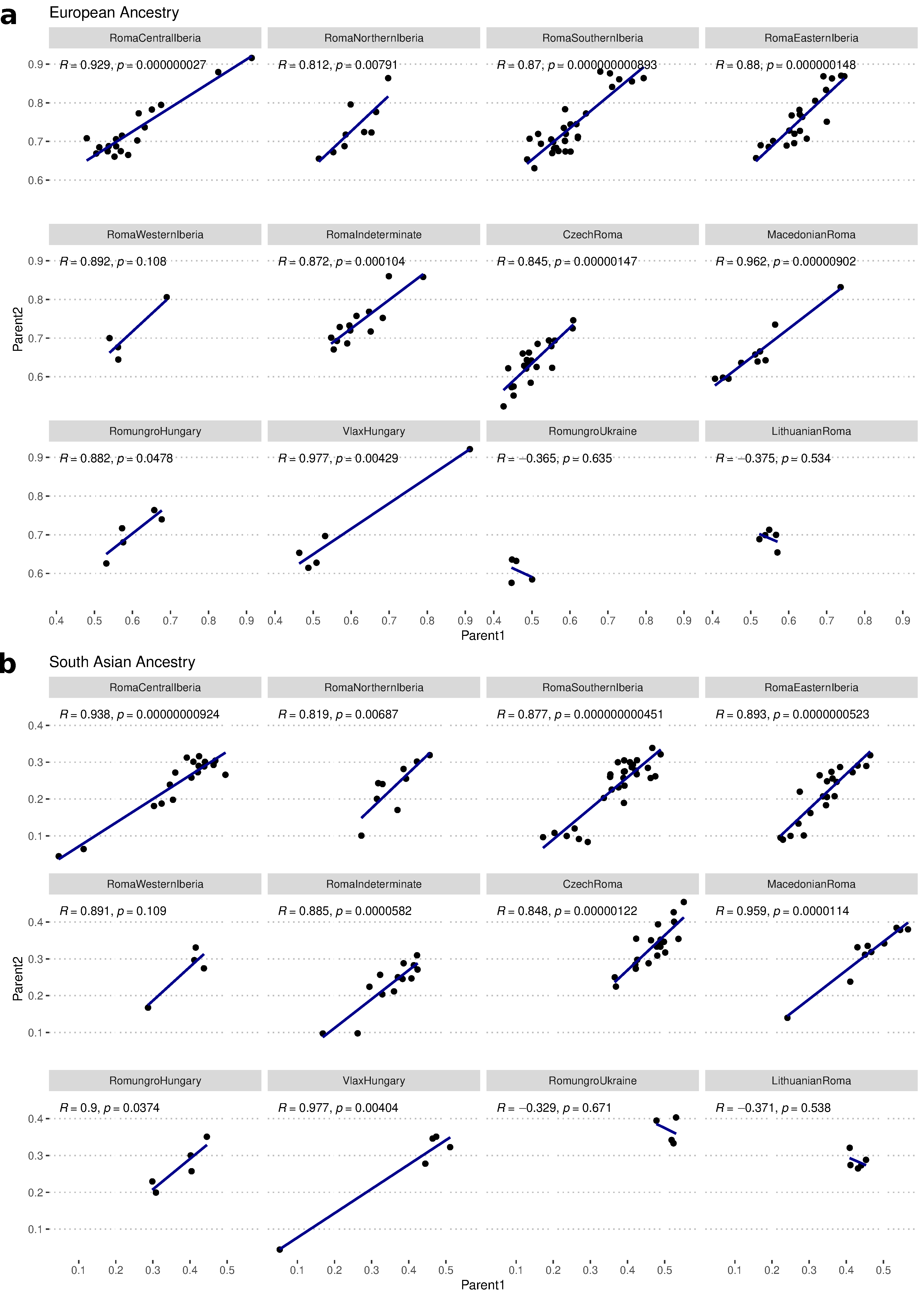
**

**Supplementary Figure 25** Assortative mating analysis results for each Roma population.

*(a) Assortative mating test for the European ancestral component. (b) Assortative mating test for the South Asian ancestral component.*

**
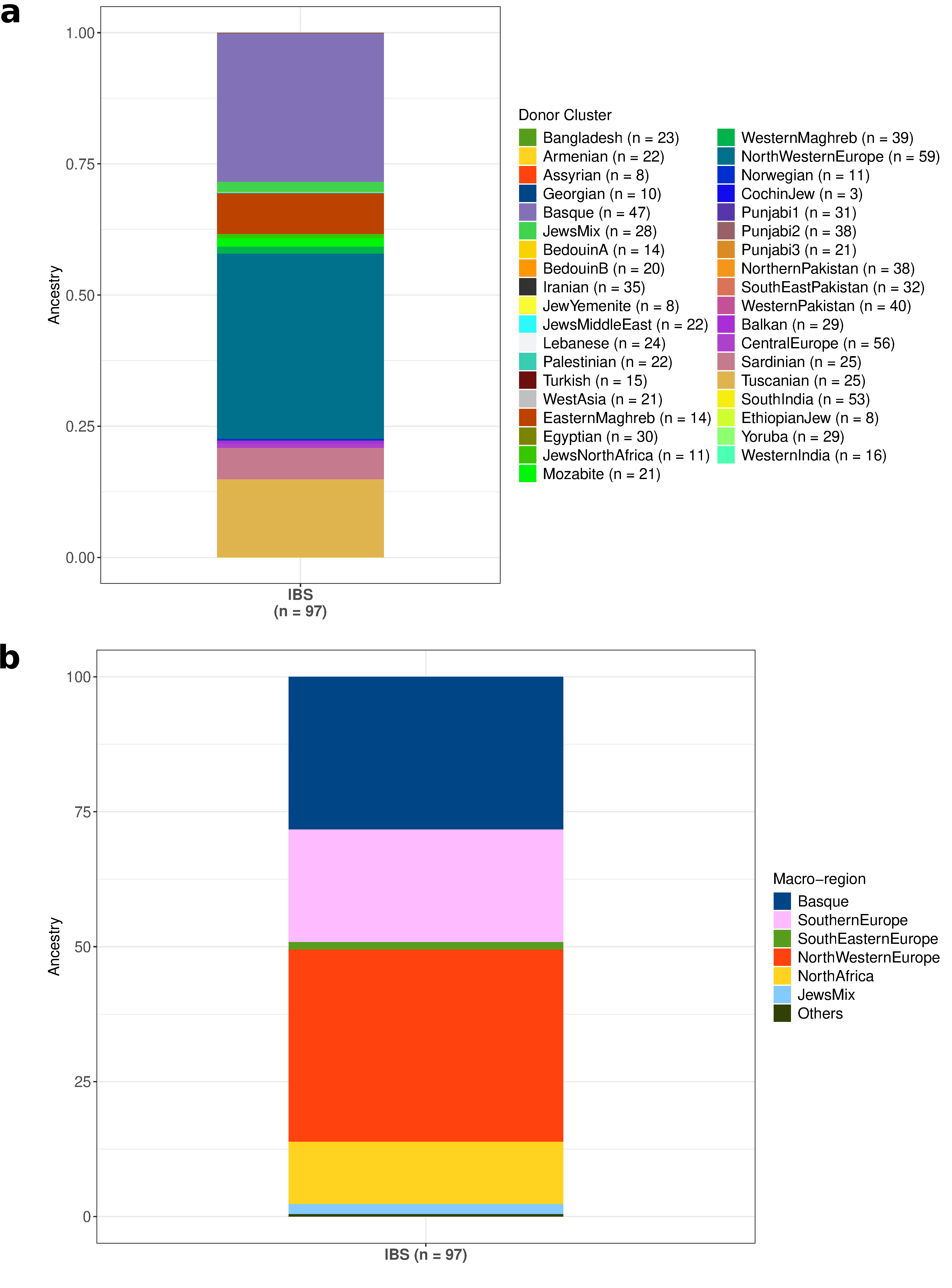
**

**Supplementary Figure 26 R**esults of the NNLS analysis with IBS as the recipient cluster.

*(a) NNLS analysis by genetic cluster, with results grouped by donor cluster. (b) NNLS analysis by genetic cluster, with results grouped by donor macro-region.*

*
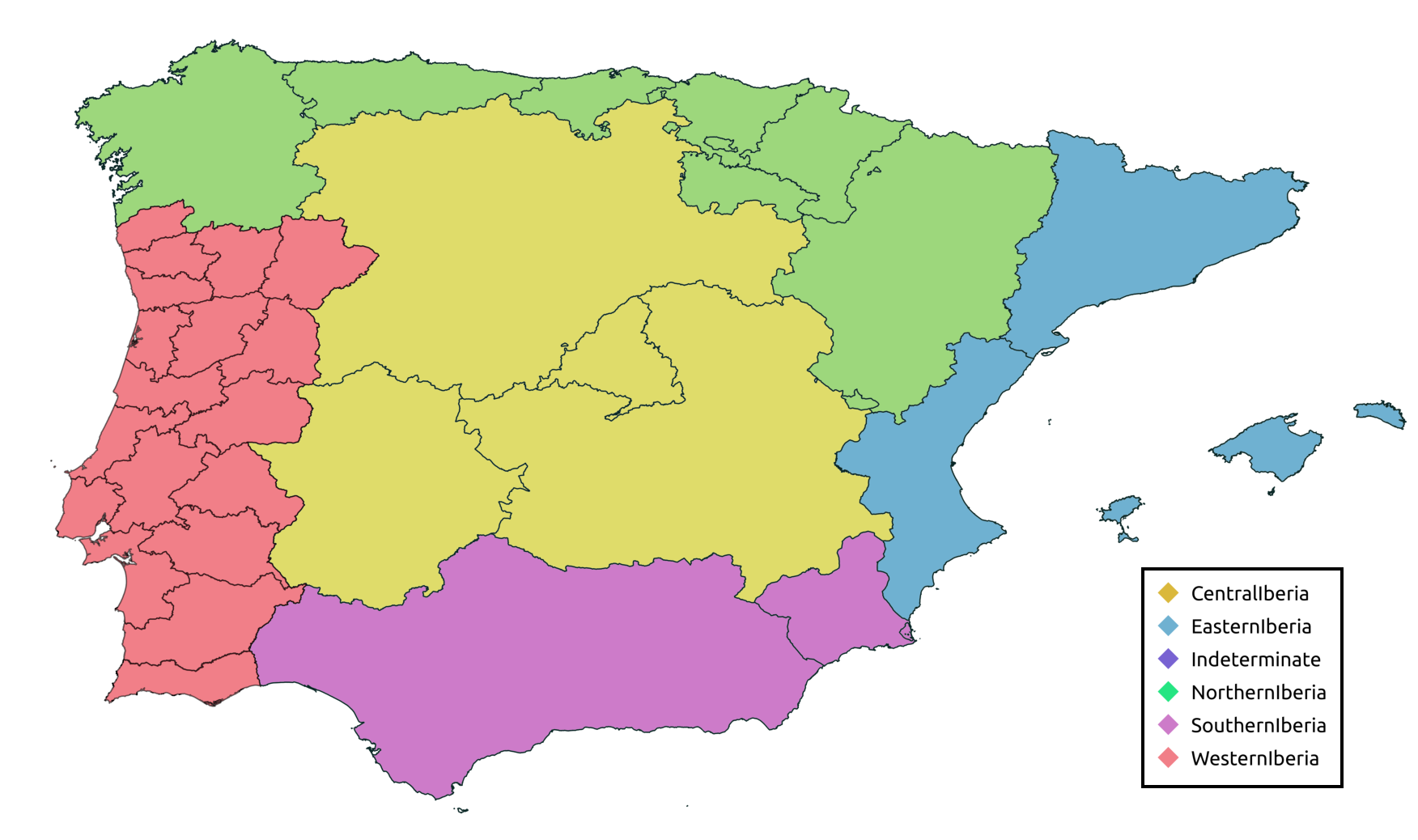
*

***Supplementary Figure 27 Distribution of the Iberian Roma samples.***

*Distribution of the Iberian Roma individuals based on their grandparents’ birthplace. The colours represent the macro-regions used for classifying the Iberian Roma within the Iberian Peninsula.*

***Supplementary Notes***

***Supplementary Note 1 IBD sharing patterns.***

The length and number of shared IBD segments between populations provide insights into the timing of admixture events and help identify trends in changing levels of endogamy. In this case, the characterization of between-population shared IBD segments is used as an indicator of admixture level over time, while the IBD segments shared within the population are a proxy for the population size and inbreeding level. We expressed shared IBD segments in three categories of comparison: 1) within-population, which are the IBD shared between individuals from the same Roma group; 2) Roma-nonRoma, the IBD shared between Roma and non-Roma individuals; 3) between-Roma, the IBD shared between individuals of different Roma groups. The length categories were assigned following an approximation (Baharian et al. 2016; Harris et al. 2018; Castro e Silva et al. 2022) which can be used to infer when the IBD segments were formed based on their length:

*E(generations ago) ≅ 3/(2I); I = IBD segment length in morgans*

Using this approximation we selected four periods: 1) Arrival into Europe ∼< 1100 CE (segments of 0-4 cM) (Fraser 1992; Kenrick 2007); 2) out-of-Balkans and arrival into Iberian peninsula ∼ 1100–1570 CE (segments of 4-8 cM) (Fraser 1992; Kenrick 2007); 3) second out-of-Balkans waves ∼ 1570–1800 CE (segments of 8-16 cM) (Achim 2004; Marushiakova and Popov 2010); 4) ending of slavery and contemporary period ∼> 1800 (segments >16 cM) (Achim 2004; Greenberg 2010). Where 4 cM corresponds to circa 940 years ago (37.5 generations ago * 25 years per generation), and 16 cM corresponds to circa 230 years ago (9.37 generations ago * 25 years per generation). The average number of IBD segments and the average length of shared IBD were then calculated for each length category, by dividing these averages, one at a time, by the product of the sample sizes of the 2 populations being compared, as in Castro e Silva et al. (2022). Two-sided and one-sided Wilcoxon tests with Bonferroni correction were then computed to test for significance in the differences in shared IBD numbers within and between populations.

***Supplementary Note 2 Distribution of European Local Ancestry tracts.***

The measurement of LAI tract lengths of European ancestry was performed on the RFmix output files generated for the ANCESTOR (Zou et al. 2015a, b) analysis. Measurement was performed using AncestryLength.rb, a script designed to measure the length of segments in the chromosomes. This measurement is calculated using the formula:

*Ending Position of the switch ending point – Starting Position of the switch starting point*

Here, the 'switch' refers to the SNP where the ancestry changes for a given haplotype. The switch points are identified as the first and last SNP of the segment within a certain random forest window. AncestryLength.rb can iterate through multiple ancestries and individuals. It was run with two settings: --hap, which omits haplotype information (needed when considering segments of the two haplotypes as part of the same individual), and --MB, which converts the tract length to MegaBases. The tracts were divided in categories following the same criteria used in the IBD test, assuming that 1 MB = 1 cM. We grouped the segments into six categories, where the longer segments are divided into three additional categories: 16-32 MB, 32-64 MB, >64 MB, which correspond to time periods going from circa 1800 CE until the last generation. The mean tract length and the number of tract length by length category were computed for each Roma group. One-sided Wilcoxon tests with Bonferroni correction were then computed to test for significance in the differences.

***Supplementary Note 3 Genetic variation and geographic distribution.***

To assess the relationship between the inferred proportion of each ancestry component (summary of CHROMOPAINTER copying vectors obtained with NNLS) and the geographic distribution (Longitude and Latitude) of Roma individuals, Pearson's correlation tests were performed. Specifically, correlation tests were conducted for each ancestry component with longitude and latitude. The correlation coefficient (r), t-value, p-value, and 95% confidence intervals were calculated and tabulated for each test. The results were used to evaluate the strength and direction of the linear associations. For each individual we used the geographic coordinates of the principal city within their region. In the case of the “Indeterminate” group, coordinates were assigned to the geographical centre of the Iberian Peninsula. Last, for non-Iberian Roma groups we assigned the coordinates of the geographic centre of their respective countries of origin.

***Supplementary Note 4 Local Ancestry Inference.***

The local ancestry inference was estimated using the software RFMix v2 (Maples et al. 2013) with the -e switch set to perform 5 iterations of the expectation-maximization optimisation of the model. The analysis was conducted with two (European and Punjabi) parental populations, analysing both the Roma and 10 additional European individuals to provide a baseline of comparison. For the analysis, 58 individuals were used as a reference for each component. The European component was selected from Spanish (IBS), Czech, Croatian, Greek, Hungarian, and Romanian populations, while the Punjabi component came from Punjabi (PJL). To measure the North African ancestry component in the IBS, an analysis was conducted using two parental populations (European and North African), including all IBS individuals and 59 reference individuals for each component. The European component was represented by Basques from France, French, Central Europeans (CEU), and Tuscans (TSI), while the North African component was represented by Moroccans, Libyans, Algerians, and Tunisians.

**References**

Achim V (2004) The Roma in Romanian History. Central European University Press

Baharian S, Barakatt M, Gignoux CR, et al (2016) The Great Migration and African-American Genomic Diversity. PLOS Genetics 12:e1006059. https://doi.org/10.1371/journal.pgen.1006059

Castro e Silva MA, Ferraz T, Couto-Silva CM, et al (2022) Population Histories and Genomic Diversity of South American Natives. Molecular Biology and Evolution 39:msab339. https://doi.org/10.1093/molbev/msab339

Fraser AM (1992) The Gypsies (The Peoples of Europe). Blackwell Pub.

Greenberg J (2010) Report on Roma Education Today: From Slavery to Segregation and Beyond. Colum L Rev 110:919–1001

Harris DN, Song W, Shetty AC, et al (2018) Evolutionary genomic dynamics of Peruvians before, during, and after the Inca Empire. Proceedings of the National Academy of Sciences 115:E6526–E6535. https://doi.org/10.1073/pnas.1720798115

Kenrick D (2007) Historical Dictionary of the Gypsies (Romanies). Scarecrow Press

Maples BK, Gravel S, Kenny EE, Bustamante CD (2013) RFMix: A Discriminative Modeling Approach for Rapid and Robust Local-Ancestry Inference. The American Journal of Human Genetics 93:278–288. https://doi.org/10.1016/j.ajhg.2013.06.020

Marushiakova E, Popov V (2010) Gypsy/Roma European migrations from 15th century till nowadays. In: Proceedings of International Conference “Romani Mobilities in Europe: Multidisciplinary Perspectives. pp 126–139

Zhou Y, Browning SR, Browning BL (2020) A Fast and Simple Method for Detecting Identity-by-Descent Segments in Large-Scale Data. The American Journal of Human Genetics 106:426–437. https://doi.org/10.1016/j.ajhg.2020.02.010

Zou JY, Halperin E, Burchard E, Sankararaman S (2015a) Inferring parental genomic ancestries using pooled semi-Markov processes. Bioinformatics 31:i190–i196. https://doi.org/10.1093/bioinformatics/btv239

Zou JY, Park DS, Burchard EG, et al (2015b) Genetic and socioeconomic study of mate choice in Latinos reveals novel assortment patterns. Proceedings of the National Academy of Sciences 112:13621–13626. https://doi.org/10.1073/pnas.1501741112
